# Anti-Hepatocarcinoma Activity and Mechanism of Isosendanin and Its Novel Structural Analogues Isolated from the Bark of *Melia azedarach* L.: In Vitro and In Vivo Studies

**DOI:** 10.3390/antiox15050562

**Published:** 2026-04-29

**Authors:** Yuanyuan Huang, Erjian Gao, Quan Liu, Jingquan Yuan, Yanchun Wu, Wei Wang, Xiaoping Rao

**Affiliations:** 1 Institute of Advanced Carbon Conversion Technology, Huaqiao University, Xiamen 361021, China; yuanyhuang@yeah.net (Y.H.);; 2 Fujian Provincial Key Laboratory of Biomass Low-Carbon Conversion, Huaqiao University, Xiamen 361021, China; 3 College of Chemical Engineering, Huaqiao University, Xiamen 361021, China; 4 Institute of Traditional Chinese and Zhuang-Yao Ethnic Medicine, Guangxi University of Chinese Medicine, Nanning 530200, China

**Keywords:** *Melia azedarach* L., anti-hepatocarcinoma, limonoids, Isotoosendanin, ROS, oxidative stress, signaling pathways

## Abstract

*Melia azedarach* L. is a plant known for its traditional medicinal uses. Limonoids (triterpenes), which have a wide range of pharmacological effects, are the most critical active ingredients; however, their potential effects on liver cancer remain to be further explored. In this study, seven limonoids were isolated from the bark of *Melia azedarach*, including two new compounds, 11α-hydroxy-12-Oxo-Meliarachin I (**1**) and 29-Oxo-12-dehydroneoazedarachin D (**3**), along with five known compounds (**2**, **4–7**), to evaluate their effect on liver cancer in vitro. The results showed that compounds **1**–**7** exhibited varying degrees of inhibitory effects on Hep3B cells. Among these, compound **6**, Isotoosendanin (ITSN), displayed the most potent activity, with an IC_50_ value of 15.06 μg/mL. Mechanism studies have shown that ITSN inhibits cell proliferation and promotes apoptosis in Hep3B cells. It induces reactive oxygen species (ROS) accumulation to trigger oxidative stress injury, suppresses the activation of the MAPK and PI3K/AKT signaling pathways, further activates the p53 pathway to induce cell cycle arrest, and ultimately initiates the apoptotic cascade. ITSN can also inhibit tumor growth in immunodeficient mice receiving allogeneic transplantation. In summary, we systematically studied the limonoids in the bark of *Melia azedarach* and elucidated the anti-hepatocarcinoma activity of ITSN in vitro and in vivo, providing promising evidence for its potential use as a natural active ingredient in the prevention and treatment of cancer.

## 1. Introduction

Hepatocellular carcinoma (HCC) ranks as the sixth most common cancer and the third leading cause of cancer-related deaths worldwide [1]. China is not only a country with a high prevalence of HCC, but it also bears the heaviest disease burden of this malignancy globally, accounting for over 40% of all HCC cases across the world [2]. At present, the diagnosis and treatment of HCC is characterized by its insidious onset in the early stage, rapid progression, refractory treatment, and an extremely poor prognosis [3]. Although drugs and treatment regimens for HCC patients are continuously updated and optimized, they only exert limited effects on the survival of patients with advanced HCC [4], and their therapeutic efficacy is limited due to drug resistance, toxicity, and adverse effects [5]. For example, sorafenib has a good therapeutic effect on patients with advanced HCC in a systematic treatment trial, and the survival period has been extended by about 3 months. Unfortunately, due to drug resistance within 6 months, only a few patients can obtain significant benefits from sorafenib treatment. Moreover, its toxic side effects further limit its clinical value [6]. Therefore, it is very important to carry out research on alternative strategies for the prevention and treatment of liver cancer and to explore the potential of naturally derived compounds in combination with other drugs to treat the disease. In this context, plant-derived triterpenoids are increasingly being developed as promising candidates owing to their varied biological activities, low toxicity and low cost [7,8].

*Melia azedarach* L. is a plant of the genus *Melia* belonging to the family Meliaceae. It is mainly found in China, Japan, South Korea, India, Australia, and other locations. It is also cultivated in Europe and the United States [9]. In China, it is mainly distributed in Sichuan, Hunan, Fujian, Guangdong, Guangxi, Hebei, etc. [10]. The use of *Melia azedarach* has long been recorded in traditional medical books. Its bark, leaves, flowers, fruits and seeds can be used as medicines: the bark is cold and toxic, effective for killing parasites and treating ringworm; the leaves clear heat, dry dampness, kill insects, and relieve itching; the fruit promotes Qi circulation, alleviates pain, and expels parasites; the flowers clear heat, remove dampness, and relieve itching, etc. It is clinically used to treat ascariasis, enterobiasis, and dermatophytosis [11,12]. Chemical studies have shown that *Melia azedarach* contains various classes of secondary metabolites such as limonoids, sesquiterpenes, oleanane triterpenoids, dammarane triterpenoids, steroids, lignans, flavonoids, and other chemical components. Limonoids are considered to be the characteristic chemical constituents and the main pharmacodynamic material basis of *Melia azedarach*. Due to the oxidation and rearrangement of their skeletons, these plants have formed a variety of structural characteristics and have shown a series of important pharmacological results, such as analgesic, antioxidant, antimalarial, anticancer and other effects. Therefore, in the field of chemistry, *Melia azedarach* has become an important target molecule [13,14].

In cancer cells, the level of intracellular reactive oxygen species (ROS) is critical for signal transduction. At low to moderate levels, ROS can promote the proliferation, migration and invasion of cancer cells. In contrast, at high levels, ROS cause oxidative damage to proteins, nucleic acids, lipids, cell membranes and organelles and trigger cell death through multiple mechanisms [15]. Epoxyazadiradione induces the production of ROS in HNSCC cell lines, thereby downregulating the expression levels of Bcl-xL, Bcl-2, and MMP9 and ultimately promoting tumor cell apoptosis [16]. Treatment with nimbolide increases the level of ROS in cells, leading to endoplasmic reticulum stress and DNA damage. This eventually induces apoptosis in NSCLC cells [17]. Furthermore, nimbolide can effectively block the growth and metastasis of pancreatic ductal adenocarcinomas by inhibiting the expression and activity of SOD2 and suppressing the activation of the PI3K/Akt signaling pathway [18]. When liver cancer cells were treated with Walsuronoid B, ROS and p53 promoted each other’s production, which significantly triggered G2/M phase arrest and mitochondrial and lysosomal apoptosis [19]. This suggests that limonoid components exert potential anticancer bioactivities by inducing the production of ROS, causing oxidative stress damage and triggering cell proliferation inhibition, cell cycle arrest, and apoptotic pathways.

In this study, we systematically studied the chemical constituents of *Melia azedarach*. Seven limonoids were isolated from *Melia azedarach*, including two new compounds and five known compounds. The anti-HCC activity of compounds **1**–**7** was evaluated by MTT assay using Hep3B cells as a model. To further explore the anti-HCC mechanism of these constituents, ITSN, which exhibited the strongest cytotoxic activity, was selected as the representative compound for mechanistic studies. The results showed that ITSN induced the generation of ROS and caused oxidative stress. Meanwhile, cell apoptosis was analyzed via staining assays and flow cytometry. To uncover the underlying molecular mechanisms, key signaling pathways were screened via cell transcriptomics, and the effects of ITSN on the MAPK, PI3K/AKT, cell cycle, and apoptosis pathways were evaluated. Among these pathways, the MAPK pathway was identified as the core pathway involved in ITSN-mediated therapy against HCC. On the other hand, ITSN can also inhibit tumor growth in immunodeficient mice receiving allogeneic transplantation. In summary, ITSN is a natural product that exerts anti-HCC effects through multiple pathways, supporting its potential as a candidate drug for liver cancer treatment.

## 2. Materials and Methods

### 2.1. Materials

The detailed information on the main apparatus, reagents, and medicinal materials used in this study is provided in Supplementary Material S1.

### 2.2. Extraction and Isolation

The air-dried powdered bark of *Melia azedarach* (45 kg) was extracted twice (each time for 2 h) with 95% EtOH and once with 85% EtOH. Evaporation of the EtOH under reduced pressure afforded the extract (2.5 kg). The residue was suspended in H_2_O and successively partitioned with PE (3 × 2 L), DCM (3 × 2 L), and EtOAc (3 × 2 L). The EtOAc fraction (290 g) was subjected to CC over silica gel (100–200 mesh), and this was eluted with CH_2_Cl_2_-MeOH (from 100:0 to 0:1, *v*/*v*) to yield ten fractions, Fs. A to Fs. J. Fs. F was subjected to MCI column chromatography elution with MeOH-H_2_O (10:90 to 100:0, *v*/*v*), yielding seven fractions, Fs. F-1 to Fs. F-7. Fs. F-4 was purified using Sephadex LH-20 CC, eluted with MeOH to give seven fractions, Fs. F-4-1 to Fs. F-4-7. Fs. F-4-1 was fractioned on an ODS C_18_ column eluted with MeOH-H_2_O (10:90 to 100:0, *v*/*v*) to afford seven fractions, Fs. F-4-1-1 to Fs. F-4-1-7. Fs. F-4-1-3 was separated by preparative HPLC (MeOH-H_2_O 40:60, 3 mL/min, *v*/*v*) to yield compounds **1** and **2**. Fs. F-4-1-4 was chromatographed by preparative HPLC using MeOH-H_2_O (52:48, *v*/*v*) to yield compound **3**. Fs. G was loaded on an MCI column chromatograph, and this was eluted with MeOH-H_2_O (10:90 to 100:0, *v*/*v*) giving seven fractions, Fs. G-1 to Fs. G-7. Fs. G-4 was subjected to CC over silica gel (200–300 mesh), eluted with CH_2_Cl_2_-MeOH (from 50:0 to 0:1, *v*/*v*) to yield seven fractions, Fs. G-4-1 to Fs. G-4-7. Fs. G-4-1 was subjected to CC over silica gel (200–300 mesh) eluted with a stepwise gradient of PE-EtOAc (from 2:1 to 1:1, *v*/*v*) to afford compound **4**. Fs. G-4-2 was purified using Sephadex LH-20 CC, eluted with MeOH to give eight fractions, Fs. G-4-2-1 to Fs. G-4-2-8. Fs. G-4-2-2 was fractioned on an ODS C_18_ column, eluted with ACN-H_2_O (10:90 to 100:0, *v*/*v*) to yield six fractions, Fs. G-4-2-2-1 to Fs. G-4-2-2-6. Fs. G-4-2-2-3 was separated by preparative HPLC (ACN-H_2_O 20:80, 3 mL/min, *v*/*v*) to yield compounds **5** and **6**. Fs. G-4-2-2-4 was isolated by preparative HPLC (ACN-H_2_O 20:80, 3 mL/min, *v*/*v*) to yield compound **7**.

### 2.3. Hep3B Cell Culture

Hep3B cells, a human hepatocellular carcinoma line, were purchased from the Cell Bank of the Chinese Academic of Science (Shanghai, China). Hep3B cells were cultured in DMEM medium containing 10% fetal bovine serum (FBS), 1% antibiotics penicillin–streptomycin, and placed in a 5% CO_2_ cell incubator at 37 °C.

### 2.4. Cell Viability Assay

The cytotoxicity of compounds **1**–**7** was evaluated using the MTT procedure with Hep3B cells. The cells in good condition during the logarithmic growth phase were seeded at a density of 1 × 10^5^ cells/mL (100 μL/well) in a 96-well plate. After 24 h of incubation, the cells were treated with different concentrations of compounds **1**–**7** for 48 h. Finally, 10 μL of MTT solution at a concentration of 5 mg/mL was added to each well, after which the plate was incubated for 4 h. Then, the supernatant was removed, and 150 μL of DMSO was put into each well. The absorbance at 570 nm was then measured using a microplate reader (Thermo Fisher Scientific, Waltham, MA, USA).

### 2.5. RNA-Seq

Cell samples were sent to Novogene Co., Ltd. (Beijing, China) for RNA-Seq analysis. Total RNA was extracted using TRIzol reagent (Invitrogen by Thermo Fisher Scientific, Carlsbad, CA, USA) after the cells were treated with different concentrations of ITSN (0, 2.5, 5, and 10 μg/mL) for 48 h. The integrity and purity of the RNA were assessed using an Agilent 5400, NanoDrop 2000 Spectrophotometer, and an Agilent 5400 device was utilized to determine purity (Agilent Technologies, Santa Clara, CA, USA). Then, the RNA libraries were sequenced on the Illumina platform. Raw sequencing data were subjected to quality control using fastp software (v0.20.1). The high-quality clean reads were then aligned to the human reference genome GRCh38 using HiSat 2 (v2.2.1). Novel gene prediction was performed with StringTie (v2.2.3). Subsequently, the expression levels of genes were quantified based on featureCounts (v2.0.6). Differentially expressed genes (DEGs) were analyzed using DESeq2 software (version 1.42.0). Genes with *p*-adjust ≤ 0.05 and |log2FoldChange| ≥ 1 were considered significantly differentially expressed. Ontology (GO) and Kyoto Encyclopedia of Genes and Genomes (KEGG) enrichment analyses were further conducted to investigate the biological functions and key signaling pathways of differentially expressed genes.

### 2.6. ROS Level Analysis

Hep3B cells were seeded into 6-well plates and treated with ITSN at concentrations of 0, 2.5, 5, and 10 μg/mL for 48 h. Following incubation, the cells were loaded with DCFH-DA (final concentration was 10 μM) and incubated in the dark for an additional 20 min. After DCFH-DA incubation, the supernatant was discarded, and cells were washed with cold phosphate-buffered saline (PBS) twice to remove the unbound probe. The fluorescence signals of intracellular ROS were observed and captured using a DMI8 inverted fluorescence microscope for staining analysis (Leica, Wetzlar, Germany). The relative ROS level was quantified by analyzing the fluorescence intensity of the captured images using ImageJ analysis software (Java 8). The relative ROS level was normalized to the control group (0 μg/mL ITSN treatment group).

### 2.7. Hoechst 33342 Staining Analysis

ITSN was administered to Hep3B cells at concentrations of 0, 2.5, 5, and 10 μg/mL for 48 h in 6-well culture plates. The cells were stained using Hoechst 33342 reagent for 20 min. After staining, the supernatant was discarded, and the cells were washed with PBS twice. The apoptotic status was observed and photographed using a DMI8 inverted fluorescence microscope (Leica, Wetzlar, Germany). The fluorescence intensity was further analyzed by ImageJ software (Java 8), and the degree of apoptosis was compared with that of the untreated control group.

### 2.8. Annexin V-FITC/PI Assay

ITSN was administered to Hep3B cells at concentrations of 0, 2.5, 5, and 10 μg/mL, followed by 48 h of incubation. Cells were digested using 0.25% Trypsin-EDTA Solution (with Phenol Red) and then harvested, centrifuged, and washed twice with PBS. The cells were then resuspended in binding buffer, stained with Annexin V-FITC and propidium iodide (PI), respectively, and incubated at room temperature in the dark for 5 min. The total apoptosis rate was analyzed using BD LSR Fortessa (Becton, Dickinson and Company, Franklin Lakes, NJ, USA). Four quadrants were established via flow cytometric analysis: Q1 (necrotic cells), Q2 (late apoptotic cells), Q3 (early apoptotic cells), and Q4 (viable cells). The total apoptosis rate was calculated as the combined proportion of Q2 and Q3.

### 2.9. Western Blot Analysis

Cells lysis was performed using a mixture of protease inhibitor-containing RIPA lysates, sonicated at 200 W for 5 s, and repeated three times to rupture the cell membranes. The lysate samples were incubated on ice for 30 min to lyse the nuclei, after which they were centrifuged at 12,000 rpm for 15 min at 4 °C. The total protein content was quantified using a BCA kit. Total protein extracts were mixed with SDS-PAGE Loading Buffer (5×) (with DTT) and heated in a constant-temperature metal bath for 10 min to achieve complete protein denaturation. The protein samples were resolved on 10% SDS-PAGE gels and transferred onto polyvinylidene fluoride (PVDF) membranes (Merck Millipore, 0.45 μm, Darmstadt, Germany). The membranes were blocked with rapid blocking solution for 10 min to eliminate non-specific binding, followed by three washes with 1× TBST (5 min each). Thereafter, the membranes were incubated with diluted primary antibodies at 4 °C overnight. After removal of the primary antibodies, the membranes were washed three times with 1× TBST. Then, the membranes were incubated with corresponding secondary antibodies on a shaker at room temperature for 1 h. After washing thoroughly with 1× TBST again, protein bands were detected using an ECL ultra-sensitive chemiluminescent substrate and visualized with a ChemiDoc MP imaging system (Bio-Rad ChemiDoc XRS+, Hercules, CA, USA). Band intensities were quantified using ImageJ software (Java 8) [20]. Original unedited Western blot results are available in Supplementary Material S2.

### 2.10. Animals Experimental Design

Twenty-four SPF healthy male BALB/c nude mice, 4 weeks old and weighing 16 ± 2 g, were provided by Jiangsu Huachuang Sino Pharmaceutical Technology Co., Ltd. (Taizhou, China) (license number: SCXK (Su) 2020-0009). All mice were housed at the Animal Experiment Center of Guangxi University of Chinese Medicine (license number: SYXK (Gui) 2024-0004) and were kept under standard controlled environmental conditions (temperature: 23 ± 3 °C; relative humidity: 50 ± 10%; light/dark cycle: 12 h) and had ad libitum access to a well-balanced commercial diet and water throughout the study. The mice were acclimated for one week. All animal experiments were performed in accordance with the protocol approved by the Experimental Animal Ethics Committee of Guangxi University of Chinese Medicine (Approval No.: GXTCMU-EC KS20250000-144). Hep3B cells (5 × 10^6^) were suspended in a 200 μL mixture of PBS and Matrigel (HG) at a ratio of 1:1 and then injected subcutaneously into the right armpit of each mouse.

The mice were split into four groups at random, six mice in each group, when tumors were visible, based on the experimental requirements: control group (2.5% sodium carboxymethyl cellulose solution, i.g.), ITSN low-dose group (20 mg/kg, i.g.), ITSN high-dose group (40 mg/kg, i.g.), as well as positive control group (5-Fu, 20 mg/kg, i.p.). Drug administration was performed once daily for 12 consecutive days after the tumor volume attained 100 mm^3^. Every three days, the tumor size and body weight of the mice were documented. Following the completion of the experiment, the mice were anesthetized with isoflurane for blood collection and then euthanized. Subsequently, their tumors and livers were excised and collected for subsequent analysis.

### 2.11. Hematoxylin–Eosin (H&E) Staining

Tumors and liver tissues were fixed in 4% paraformaldehyde for 48 h, after which they were dehydrated, paraffin embedded, sectioned and H&E stained [21].

### 2.12. Immunohistochemical Analysis

The prepared mouse tumor wax blocks were sliced, subjected to antigen repair, and incubated with primary antibody and secondary antibody. Subsequently, the tissue sections were colored by mixing them with the substrate chromogen solution, followed by staining with hematoxylin. After dehydration and sealing, quantitative analysis was performed via fluorescence microscopy [22]. Detailed methods are provided in Supplementary Material S1.

### 2.13. Statistical Analysis

The results were analyzed by one-way ANOVA (GraphPad Prism 8.0 software). Data were expressed as the mean ± standard (mean ± SD) error of at least three independent biological replicates. Differences were considered to be statistically significant at * *p* < 0.05, ** *p* < 0.01, and *** *p* < 0.001.

## 3. Results

### 3.1. Studies on the Chemical Constituents

**Figure 1:**
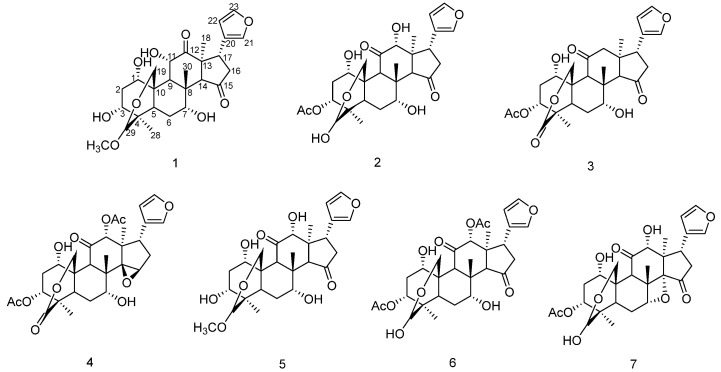
Chemical structures of compounds **1**–**7**.

We systematically studied the chemical constituents of *Melia azedarach*. Seven limonoids were isolated from the bark of *Melia azedarach*, including two new compounds, 11*α*-hydroxy-12-*Oxo*-Meliarachin I (**1**) and 29-*Oxo*-12-dehydroneoazedarachin D (**3**), along with five known compounds (**2**, **4**–**7**). The chemical structures of compounds **1**–**7** are shown in Figure 1. The NMR spectra, IR spectra, and MS spectra of the new compounds and the NMR data of all compounds are shown in Supplementary Material S3.

#### Structural Analysis of Compounds **1** and **3**

Compound **1** was isolated as a white amorphous powder. The IR (KBr) spectrum showed absorptions that could be attributed to hydroxyl (*ν*_max_ 3434 cm^−1^), methyl (*ν*_max_ 2928 cm^−1^), carbonyl (*ν*_max_ 1728 cm^−1^), and carbon–carbon double bond (*ν*_max_ 1628 cm^−1^) groups. The molecular formula was determined as C_27_H_36_O_9_ by HR-ESI-MS at *m*/*z* 505.2555 [M + H] ^+^ (Anal. Calcd.: 505.2538), which is consistent with 10 degrees of unsaturation.

**Figure 2:**
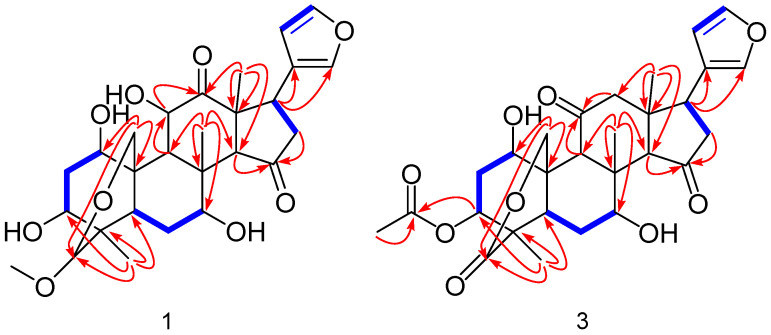
Key HMBC (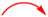) and ^1^H-^1^H COSY (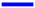) correlations of compounds **1** and **3**.

**Figure 3:**
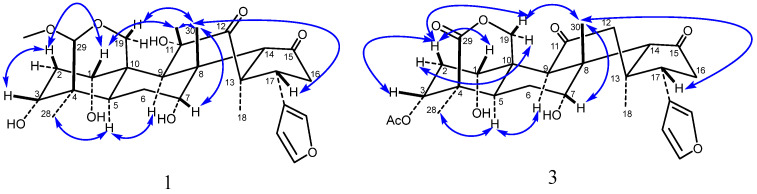
Key NOESY (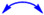) correlations of compounds **1** and **3**.

The ^1^H NMR spectrum showed four methyl singlets at *δ*_H_ 0.97 (3H, s, 28-CH_3_), 1.11 (3H, s, 30-CH_3_), 1.12 (3H, s, 18-CH_3_), and 3.33 (3H, s, 29-OCH_3_); seven oxygenated proton signals at *δ*_H_ 3.45 (1H, br s, H-3), 3.67 (1H, d, *J* = 11.5 Hz, H-19*α*), 4.02 (1H, br s, H-7), 4.30 (1H, d, *J* = 11.5 Hz, H-19*β*), 4.11 (1H, s, H-29), 4.43 (1H, dd, *J* = 11.7, 1.6 Hz, H-11), and *δ*_H_ 4.51 (1H, br s, H-1); and three hydrogen proton signals on the furan rings at *δ*_H_ 6.42 (1H, s, H-22), 7.38 (1H, s, H-21), and 7.42 (1H, s, H-23). Combined analysis of the ^13^C-NMR and DEPT 135^°^ spectrum indicated that compound **1** possesses a skeleton based on 27 carbons, including four methyl carbons *δ*_C_ 19.2 (C-30), 19.3 (C-28), 23.1 (C-18), and 55.7 (29-OCH_3_); six oxygenated carbons *δ*_C_ 60.4 (C-19), 70.6 (C-7), 73.1 (C-11), 73.4 (C-1), 76.2 (C-3), and 105.0 (C-29); four carbon signals on the furan rings *δ*_C_ 112.3 (C-22), 124.4 (C-20), 142.1 (C-21), and 143.9 (C-23), and two carbonyl carbon signals *δ*_C_ 214.3 (C-12) and 218.8 (C-15). All the proton signals were assigned to the corresponding carbons through direct ^1^H and ^13^C correlations in the HSQC spectrum. The ^1^H and ^13^C NMR spectra of compound **1** were similar to those of Meliarachin I [23], the difference being that the C-11 of compound **1** is hydroxyl, and the C-12 position is carbonyl, while for Meliarachin I, the results are the opposite. The correlation from H-9 (*δ*_H_ 2.64) to C-11 (*δ*_C_ 73.1) and H-18 (*δ*_H_ 1.12) to C-12 (*δ*_C_ 214.3), C-13 (*δ*_C_ 54.0), C-14 (*δ*_C_ 64.3), and C-17 (*δ*_C_ 39.1), according to the HMBC supported the above result. The ^1^H-^1^H COSY revealed that compound **1** possessed five fragments, H-1/H-2/H-3, H-5/H-6/H-7, H-9/H-11, H-16/H-17, and H-22/H-23, as shown in Figure 2. In addition, the HMBC spectrum (Figure 2) also showed the presence of key correlations between H-9 (*δ*_H_ 2.64), H-19 (*δ*_H_ 3.67, 4.30) and C-1 (*δ*_C_ 73.4); H-28 (*δ*_H_ 0.97) and C-3 (*δ*_C_ 76.2), C-4 (*δ*_C_ 42.6), and C-5 (*δ*_C_ 25.6); H-30 (*δ*_H_ 1.11) and C-7 (*δ*_C_ 70.6), C-8 (*δ*_C_ 43.1), C-9 (*δ*_C_ 43.5), and C-14 (*δ*_C_ 64.3); H-14 (*δ*_H_ 3.24) and C-15 (*δ*_C_ 218.7); H-29 (*δ*_H_ 4.11) and C-3 (*δ*_C_ 76.2), C-5 (*δ*_C_ 25.6), C-19 (*δ*_C_ 60.4), and C_29_-OCH_3_ (*δ*_C_ 55.7); H_29_-OCH_3_ (*δ*_H_ 3.33) and C-29 (*δ*_C_ 105.0), indicating that three hydroxyl groups were located at C-1, C-3, C-7; a carbonyl group was located at C-15; and a methoxy group was located at C-29, respectively. The relative configuration of compound **1** was determined by analyses of its NOESY data. The NOESY correlations (Figure 3) were also detected between H_3_-30*β*/H-7/H-11, H-11/H-1/H-2/H-3 and indicated the *α*-orientations of 1-OH, 3-OH, 7-OH and 11-OH, on the basis of the above results. In addition, compound **1** exhibited similar carbon chemical shifts, proton coupling constants, and NOESY correlations to those of the known compound Meliarachin I. Combined with the reported biosynthetic pathway of natural trichilin-type limonoids [14], the absolute configuration of compound **1** was therefore deduced to be 1*S*, 3*R*, 4*R*, 5*R*, 7*R*, 8*S*, 9*S*, 10*S*, 13*S*, 14*S*, 17*R*. Compound **1** was thus structurally elucidated and trivially named 11*α*-hydroxy-12-*Oxo*-Meliarachin I [(1*S*, 3*R*, 4*R*, 5*R*, 7*R*, 8*S*, 9*S*, 10*S*, 13*S*, 14*S*, 17*R*)-19, 29-epoxy-29-methoxyl-1*α*, 3*α*, 7*α*, 11*α*-tetrahydroxy-meliacane-12, 15-dione]. For spectroscopic data, see Supplementary Material S3 Table S1.

Compound **3** was obtained as a white amorphous powder. Its IR (KBr) spectrum showed absorptions of hydroxyl (*ν*_max_ 3445 cm^−1^), methyl (*ν*_max_ 2961, 2928 cm^−1^), carbonyl (*ν*_max_ 1730 cm^−1^) and a carbon–carbon double bond (*ν*_max_ 1632 cm^−1^). Its molecular formula was established as C_28_H_34_O_9_ from the HR-ESI-MS ion at *m*/*z* 515.2294 [M + H] ^+^ (Anal. Calcd.: 515.2281), consistent with 12 degrees of unsaturation.

The ^1^H NMR spectrum showed four methyl singlets at *δ*_H_ 1.04 (3H, s, 18-CH_3_), 1.11 (3H, s, 30-CH_3_), 1.21 (3H, s, 28-CH_3_), and 2.14 (3H, s, 3-OAc); five oxygenated proton signals at *δ*_H_ 3.94 (1H, m, H-7), 3.95 (1H, m, H-1), 4.68 (1H, d, *J* = 14.2 Hz, H-19*α*), 4.78 (1H, d, *J* = 14.1 Hz, H-19*β*), and 5.19 (1H, br s, H-3); and three hydrogen proton signals on the furan rings at *δ*_H_ 6.31 (1H, s, H-22), 7.43 (1H, s, H-21), and 7.32 (1H, s, H-23). The ^13^C NMR and and DEPT 135^°^ spectrum of compound **3** displayed 28 carbon signals, four methyl carbons at *δ*_C_ 19.7 (C-28), 20.1 (C-30), 21.2 (3-OAc), and 28.5 (C-18); four signals attributable to oxygen-bearing carbons at *δ*_C_ 69.2 (C-7), 71.0 (C-1), 72.5 (C-19), and 74.6 (C-3); four carbon signals on the furan rings *δ*_C_ 110.4 (C-22), 121.8 (C-20), 143.7 (C-21), and 140.4 (C-23); an ester carbonyl carbon signal *δ*_C_ 172.7 (C-29); and two carbonyl carbon signals *δ*_C_ 210.6 (C-11), 219.5 (C-15). All proton signals were assigned to the corresponding carbons through direct ^1^H and ^13^C correlations in the HSQC spectrum. The NMR data were similar to those reported for 12-dehydroneoazedarachin D [24]. The difference is that C-29 of compound **3** is carbonyl. In the HMBC spectrum, the correlation observed from H-28 (*δ*_H_ 1.21) to C-3 (*δ*_C_ 74.6), C-4 (*δ*_C_ 46.5), C-5 (*δ*_C_ 28.3), and C-29 (*δ*_C_ 172.7), together with the molecular formula, confirmed the above deduction. Inspection of the ^1^H-^1^H COSY spectrum showed fragments of H-1/H-2/H-3, H-5/H-6/H-7, H-16/H-17, and H-22/H-23 (Figure 2). In addition, the HMBC spectrum also showed the presence of key correlations between H-2 (*δ*_H_ 2.09), H-9 (*δ*_H_ 3.48), H-19 (*δ*_H_ 4.68, 4.78), and C-1 (*δ*_C_ 71.0); H-28 (*δ*_H_ 1.21) and C-3 (*δ*_C_ 74.6), C-4 (*δ*_C_ 46.5), C-5 (*δ*_C_ 28.3), and C-29 (*δ*_C_ 172.7); H-3 (*δ*_H_ 5.19), 3-OCOCH_3_ (*δ*_H_ 2.14) and 3-OCOCH_3_ (*δ*_C_ 169.0); H-30 (*δ*_H_ 1.11) and C-7 (*δ*_C_ 69.2), C-8 (*δ*_C_ 45.0), C-9 (*δ*_C_ 53.1), and C-14 (*δ*_C_ 63.1); H-9 (*δ*_H_ 3.48), H-12 (*δ*_H_ 2.32, 2.72), and C-11 (*δ*_C_ 210.6); H-14 (*δ*_H_ 3.15), H-16 (*δ*_H_ 2.60), and C-15 (*δ*_C_ 219.5), indicating that two hydroxyl groups were located at C-1 and C-7; a acetyloxy group was located at C-3; and three carbonyl group were located at C-11, C-15, and C-29, respectively (Figure 2). The NOESY correlations of H_3_-30*β*/H-19*β*/H-2, H-2*β*/H-1/H-3 and H_3_-30*β*/H-7 indicated the *α*-orientations of 1-OH, 3-OAc, and 7-OH (Figure 3). In addition, compound **3** exhibited similar carbon chemical shifts, proton coupling constants, and NOESY correlations to those of the known compound 12-dehydroneoazedarachin D. Combined with the reported biosynthetic pathway of natural trichilin-type limonoids [14], the absolute configuration of compound **3** was therefore deduced to be 1*S*, 3*R*, 4*R*, 5*R*, 7*R*, 8*S*, 9*S*, 10*S*, 13*S*, 14*S*, 17*R*. Therefore, the structure of compound **3** was established and trivially named 29-Oxo-12-dehydroneoazedarachin D [(1*S*, 3*R*, 4*R*, 5*R*, 7*R*, 8*S*, 9*S*, 10*S*, 13*S*, 14*S*, 17*R*)-19, 29-epoxy-29-Oxo-1*α*, 7*α*, -dihydroxy-3*α*-acetoxyl-meliacane-11, 15-dione]. For spectroscopic data, see Supplementary Material S3 Table S1.

Through the comparison of NMR data with previously reported spectroscopic data, five known compounds were identified as 12-hydroxyamoorastatone (**2**) [25], Meliarachin B (**4**) [23], Meliarachin I (**5**) [23], Isotoosendanin (**6**) [26], and Meliarachin F (**7**) [23].

### 3.2. Compounds ***1***–***7*** Inhibit the Proliferation of Hep3B Cells

To evaluate the cytotoxicity of compounds **1**–**7** against hepatoma cells, Hep3B cells were treated with compounds **1**–**7** at various concentrations (0, 1.875, 3.75, 7.5, 15, 30, 60, and 120 μg/mL) for 48 h. Cell proliferation and viability were determined using the MTT assay. The results indicated that compounds **1**–**7** exhibited varying inhibitory effects on Hep3B cells, with IC_50_ values of 89.19 ± 2.10, 41.64 ± 1.92, 67.23 ± 1.23, 23.86 ± 1.60, 102.56 ± 1.50, 15.06 ± 1.27, and 37.74 ± 1.15 μg/mL, respectively. Based on the above results, ITSN, with the best activity, was selected in this study for further mechanistic investigations.

### 3.3. RNA-Seq Analysis Results

**Figure 4:**
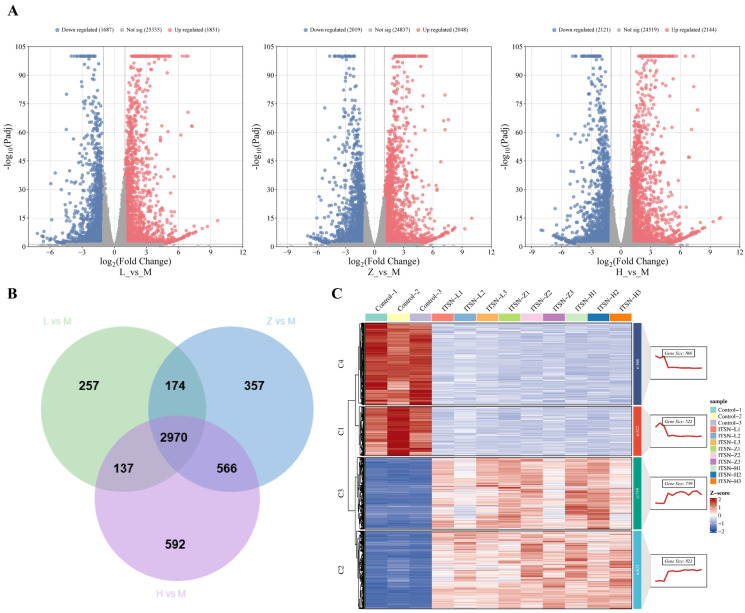

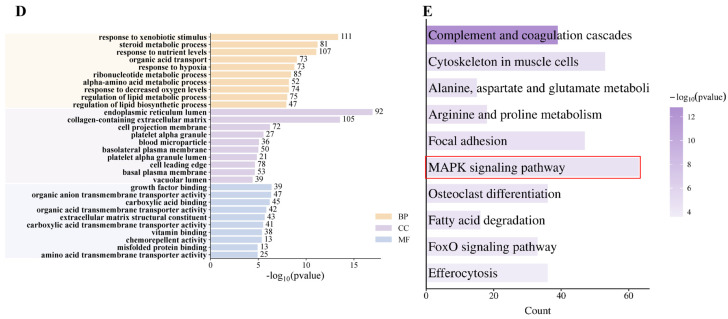
Comprehensive characterization of ITSN intervention in Hep3B cells via RNA-Seq analysis. (**A**) Volcano plot of ITSN-induced differentially expressed mRNAs in Hep3B cells; (**B**) Venn diagram of differentially expressed mRNAs in Hep3B cells after three different concentrations of ITSN intervention; (**C**) heatmap of differentially expressed mRNAs in Hep3B cells following ITSN treatment; (**D**) GO enrichment analysis of 2970 DEGs; (**E**) KEGG enrichment analysis of 2970 DEGs.

Cell transcriptomics can systematically analyze the gene expression profiles and regulatory networks in cells at the global level, screen key functional genes and signaling pathways, reveal the molecular mechanisms of life activities and disease occurrence, and provide important theoretical basis and direction guidance for subsequent mechanism research, disease diagnosis, and drug development. Results showed that a total of 3538 DEGs were identified in the ITSN-L group (2.5 μg/mL) compared with the results for the control group (M group), including 1851 upregulated and 1687 downregulated genes (Figure 4A). For the ITSN-Z group (5 μg/mL), 4064 DEGs were detected relative to those in the control group (M group), with 2048 being upregulated and 2019 downregulated (Figure 4A). In contrast, 4265 DEGs were obtained in the ITSN-H group (10 μg/mL) vs. the control group (M group), including 2144 upregulated and 2121 downregulated genes (Figure 4A). Overlap analysis of DEGs across the three comparison groups was performed, and Venn diagram results showed 2970 shared DEGs (Figure 4B). To clarify the underlying mechanism by which ITSN exerts its effects on the HCC, GO and KEGG pathways enrichment analysis were performed on these 2970 overlapping DEGs. As shown in the bar plot (Figure 4D), the top enriched biological processes included the response to xenobiotic stimulus (111 genes), the steroid metabolic process (107 genes), and the response to nutrient levels (81 genes), suggesting a profound impact on cellular stress and metabolic pathways. In the cellular component category, the collagen-containing extracellular matrix (105 genes) was the most significantly enriched term, implying that ITSN may modulate extracellular matrix remodeling. Additionally, molecular function analysis revealed significant enrichment in growth factor binding, organic anion transmembrane transporter activity, and carboxylic acid transmembrane transporter activity, all of which are critical for signal transduction and nutrient transport. These findings collectively indicate that ITSN exerts its biological effects in Hep3B cells by regulating multiple key biological processes, cellular components, and molecular functions. KEGG analysis revealed that the significantly differentially expressed genes were mainly enriched in the MAPK pathway, cytoskeleton in muscle cells, and other signaling pathways (Figure 4E). It can be inferred that ITSN exerts its anti-HCC effects primarily through the regulation of the MAPK signaling pathway.

### 3.4. ITSN Increases the Level of ROS

**Figure 5:**
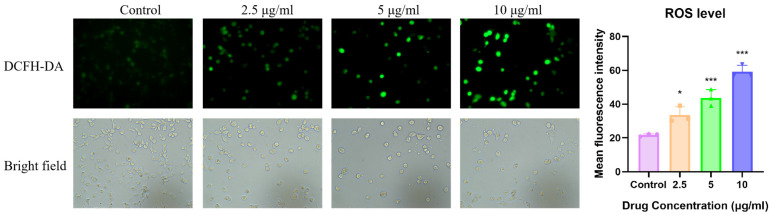
Hep3B cells were treated with ITSN for 48 h. The ROS level was measured using DCFH-DA (20×). * *p* < 0.05, *** *p* < 0.001 vs. untreated controls.

It has been reported that ROS play a dual regulatory role in the occurrence, development and treatment of cancer. An appropriate level of ROS can promote the occurrence and development of tumors. However, excessive ROS can induce oxidative stress and achieve selective elimination of cancer cells by activating apoptotic pathways, necrosis, or pyroptosis, which is also the core mechanism underlying the anti-tumor effects of traditional radiotherapy, chemotherapy, and targeted therapy [27,28]. To detect ITSN-induced oxidative stress, cells treated with ITSN were stained using the DCFH-DA probe (Figure 5). The results showed that the fluorescence of cells in the control group was weak, whereas fluorescence intensity increased in a dose-dependent manner after ITSN treatment compared with the results for the control group. This indicates that ITSN can trigger oxidative stress by inducing the production of ROS, thereby promoting cancer cell apoptosis.

### 3.5. ITSN Increased the Apoptosis of Hep3B Cells

**Figure 6:**
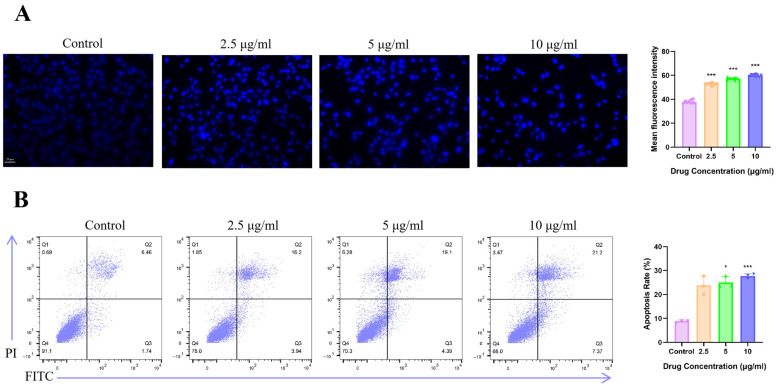

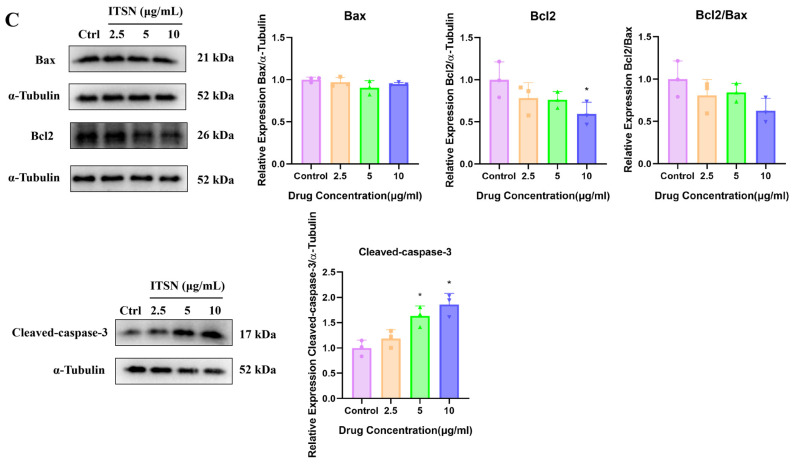
(**A**) Hep3B cells were treated with ITSN, and the morphological characteristics of apoptosis after Hoechst 33342 staining are shown (20×). (**B**) The apoptosis rate of Hep3B cells treated with ITSN was detected by Annexin V-FITC and PI staining. (**C**) Western blot analysis of apoptosis-associated proteins. * *p* < 0.05, *** *p* < 0.001 vs. untreated controls.

Hoechst 33342 staining is a widely used method for detecting apoptosis, by which the existence of apoptosis can be clearly and intuitively observed. Apoptotic cells show morphological changes and cellular shrinkage with increased membrane permeability [29], allowing more dye to enter the cells and resulting in enhanced fluorescence. As shown in Figure 6A, the viability of Hep3B cells treated with ITSN for 48 h was significantly reduced compared with that of the control group, and a large number of broken or condensed nuclei appeared in the cells, accompanied by markedly increased blue fluorescence intensity. Meanwhile, we also analyzed the effect of ITSN on apoptosis in Hep3B cells by flow cytometry. The results demonstrated that compared with the control group, the ITSN-treated groups exhibited an increase in the apoptotic cell population, reaching approximately 20.14%, 23.49%, and 28.57%, respectively (Figure 6B). These findings indicate that ITSN can induce apoptosis in Hep3B cells.

To further dissect the apoptotic program elicited by ITSN, we performed Western blot analysis to quantify the expression of core apoptotic regulators. As shown in the results (Figure 6C), treatment with ITSN for 48 h resulted in a dose-dependent downregulation of Bcl-2 and Bcl-2/Bax ratio expression, as well as the upregulation of cleaved caspase-3 expression, suggesting the activation of the apoptotic pathway.

### 3.6. Effects of ITSN on MAPK and PI3K/AKT Signaling Pathways in Hep3B Cells

Following intervention with varying concentrations of ITSN, cellular proliferation was inhibited, and apoptosis was promoted. To comprehensively explore the potential core mechanisms underlying the anti-HCC activity of ITSN, the transcriptomics results were combined and analyzed. Notably, the MAPK pathways were closely associated with tumorigenesis. Additionally, the PI3K/AKT signaling pathway also exerts a synergistic regulatory effect. Therefore, we hypothesized that these pathways may play a pivotal role in mediating the anti-HCC activity of ITSN.

**Figure 7:**
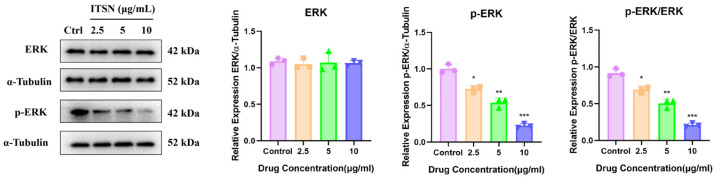

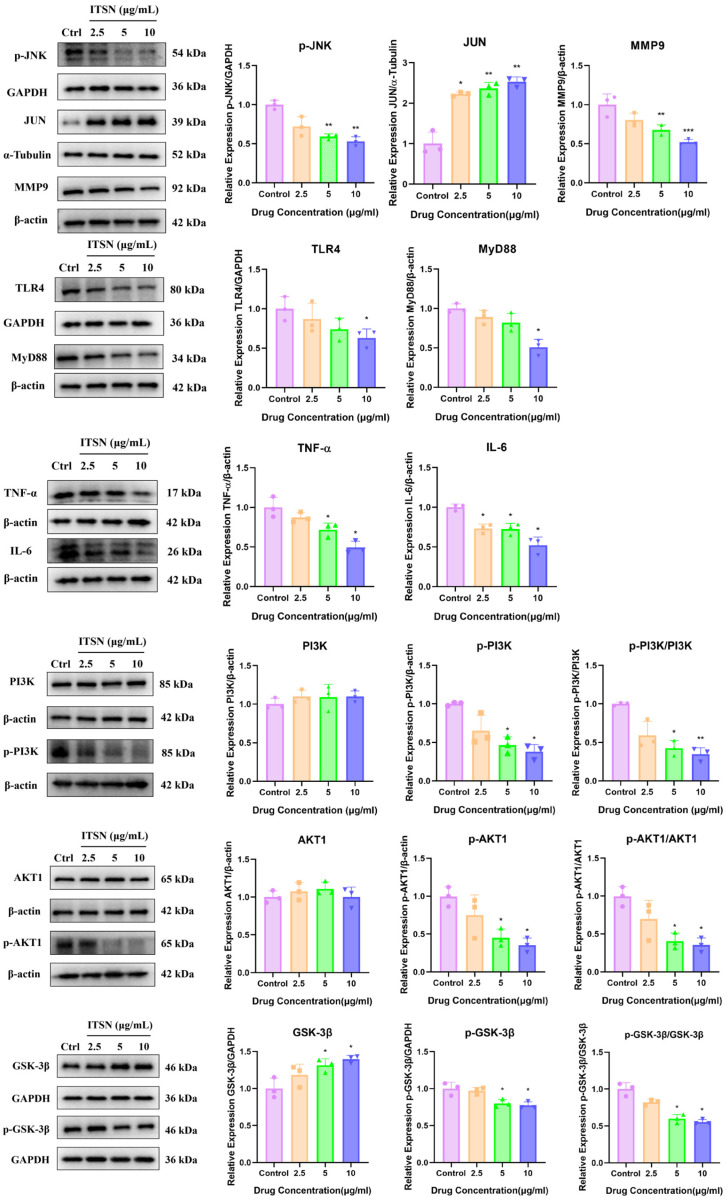
Western blot analysis of related protein expression in MAPK and PI3K/AKT in Hep3B cells. * *p* < 0.05, ** *p* < 0.01, *** *p* < 0.001 vs. untreated controls.

The MAPK and PI3K/AKT signaling pathways are critical regulators of cell proliferation, survival, apoptosis, invasion, metastasis, and drug resistance. These pathways are frequently aberrantly activated in various tumors, including hepatocellular carcinomas [30,31]. Therefore, inhibiting the activation of these two pathways in Hep3B cells can effectively exert anti-HCC effects. To test our hypothesis, we assessed the expression of relevant proteins, as well as their phosphorylated forms, in Hep3B cells. As shown in Figure 7, ITSN significantly suppressed the expression of p-ERK, p-JNK, MMP9, TLR4, MyD88, IL-6, TNF-α, p-PI3K, p-AKT1 and p-GSK-3β, while increasing the expression of JUN and GSK-3β, which suggests that ITSN can effectively inhibit the MAPK and PI3K/AKT signaling pathways (Figure 7).

### 3.7. Effects of ITSN on p53 Signaling Pathway in Hep3B Cells

**Figure 8:**
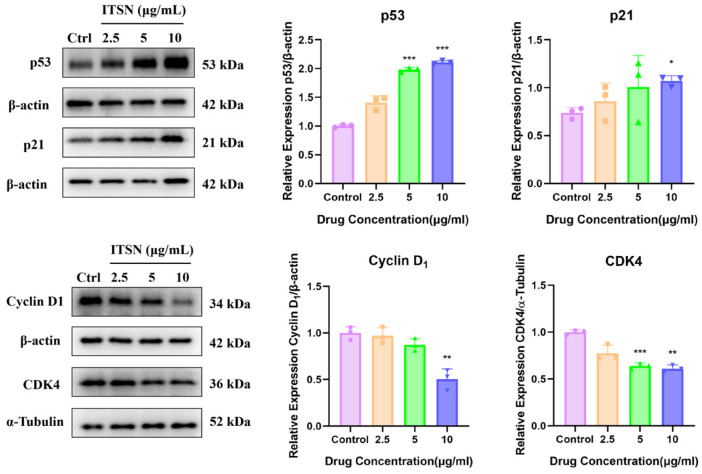
Western blot of related protein expression in p53 in Hep3B cells. * *p* < 0.05, ** *p* < 0.01, *** *p* < 0.001, vs. untreated controls.

Inducing cell cycle arrest in tumor cells can effectively inhibit their abnormal proliferation and promote apoptosis or senescence. As shown in Figure 8, ITSN significantly increased the expression of p53 and p21, and decreased the expression of CDK4 and Cyclin D1. This indicates the occurrence of abnormal cell cycle arrest.

### 3.8. ITSN Inhibits the Growth of Xenograft Tumors In Vivo

**Figure 9:**
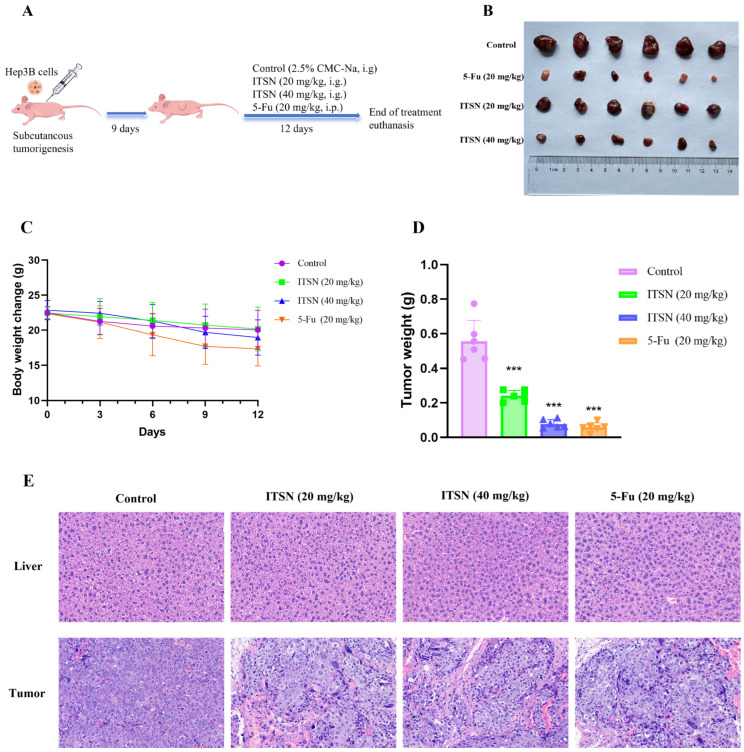

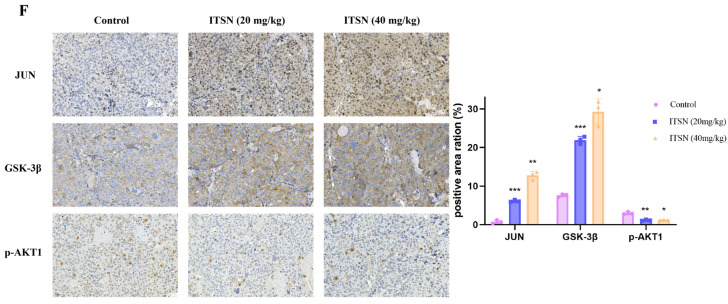
In a mouse xenograft model in vivo, ITSN suppressed tumor growth. (**A**) In vivo experimental flowchart; (**B**) images of tumors; (**C**) body weight; (**D**) comparison of tumor weight; (**E**) H&E staining of liver and tumor (20×); (**F**) IHC staining of JUN, GSK-3β, and p-AKT1 in tumor tissues (20×). * *p* < 0.05, ** *p* < 0.01, *** *p* < 0.001 vs. untreated controls. A portion of the diagram was created with BioGDP.com [32].

To evaluate the in vivo anti-tumor effect of ITSN, a BALB/c nude mouse xenograft tumor model was established. When the tumor volume reached 100 mm^3^, the mice were treated with 2.5% CMC-Na solution (i.g.), ITSN (20 mg/kg, i.g.), ITSN (40 mg/kg, i.g.), and 5-Fu (20 mg/kg, i.p.) once daily. After 12 days of treatment, the mice were euthanized, and the xenograft tumors and livers were excised (Figure 9A). During the treatment period, the body weight of the mice in the ITSN intervention group did not fluctuate significantly, while the mice in the positive drug (5-Fu) group showed significant weight loss, accompanied by progressive hunching, lethargy, and decreased appetite (Figure 9C). H&E staining of mouse liver sections showed that ITSN caused no significant liver damage (Figure 9E). Collectively, these results indicated that ITSN has no significant toxicity to mice at the tested therapeutic doses. In addition, it was evident that mice in the ITSN treatment group exhibited significantly reduced tumor weight compared with that in the control group, with tumor inhibition rates of 55.8% and 85.7%, respectively (Figure 9B,D). H&E staining showed that the cancer cells in the control group were closely arranged, the number of cells was large, the nuclear–cytoplasmic ratio was high, the boundary was clear, and more nuclear divisions were observed without necrosis. After ITSN intervention, cancer cells showed loose cell arrangement, increased vacuoles, chromatin condensation or irregular shape, reduced cell pathological mitosis, pyknosis of the nucleus, and apoptosis (Figure 9E). Immunohistochemical staining showed that the expressions of JUN and GSK-3β were increased, while the expression of p-AKT1 was decreased after ITSN intervention (Figure 9F), consistent with the results of cell Western blot analysis. Collectively, these findings demonstrate that ITSN can effectively inhibit the growth of Hep3B xenografts.

## 4. Discussion

Historically, the majority of approved therapeutic agents have been derived from natural products and their structural derivatives. Notably, approximately one-third of clinically marketed drugs are directly or indirectly sourced from natural origins [33]. Accordingly, natural-product-based drug discovery and development strategies continue to occupy a dominant and indispensable position in modern pharmaceutical research. The limonoids in *Melia azedarach* have attracted special attention due to their unique structural complexity and extensive pharmacological activities. Many compounds have shown good anticancer activity in a number of tumor models [34,35,36,37]. In this paper, we confirmed that ITSN, isolated from *Melia azedarach*, effectively inhibited HCC growth both in vitro and in vivo and revealed the multi-target and multi-pathway synergistic regulation mechanism of ITSN in the treatment of HCC.

Firstly, seven limonoids were isolated from the bark of *Melia azedarach*, among which compounds **1** and **3** were new compounds. Their chemical structures were determined on the basis of extensive spectroscopic analyses (including IR, HR-ESI-MS and NMR spectroscopy). Compounds **1**–**7** displayed differential inhibitory activities against the proliferation of Hep3B cells. Among these, compound **6** (ITSN) showed the strongest potency, with an IC_50_ value of 15.06 μg/mL. According to the results of pharmacological activity screening, we can speculate the following structure–activity relationship: hydroxyl groups at C-1 and C-7 are necessary for activity, -OAc at C-12 improves cytotoxic activity more significantly than does -OH, and -OH at C-29 is more effective than -OCH_3_. The epoxy ring at C-14/C-15 is also beneficial to the increase in activity, while the activity decreases when the C-15 is = O substituted [38].

Due to the diverse structure of natural compounds, the targets of action on tumors are also multiple [39]. Based on the significant inhibitory effect of ITSN on Hep3B cell proliferation, we systematically elucidated the multi-target mechanism of ITSN by transcriptome analysis. KEGG and GO analysis showed that the common targets shared by ITSN and HCC were significantly enriched in MAPK pathways, participating in the biological processes of tumors.

ROS are important redox signal molecules in cells, and their homeostasis is crucial for cell survival. Abnormal accumulation of ROS can trigger oxidative stress, which can induce cellular damage, senescence and apoptosis [27]. The tumor suppressor p53 is an important molecule involved in regulating cellular response to DNA damage. Studies have shown that p53 plays a role in directly interacting with ROS-damaged DNA and in facilitating cellular responses to ROS-induced DNA damage. Accordingly, when cells are exposed to severe ROS stress, the high level of DNA damage leads to persistent activation of p53 [40]. Activated p53 transcriptionally upregulates the downstream target protein p21, thereby inhibiting the activity of CDK/Cyclin complexes and inducing cell cycle arrest. Once DNA damage cannot be repaired, p53 further initiates the cellular apoptosis pathway [41]. Accumulating evidence demonstrates that many anti-tumor drugs possess the capacity to induce cell cycle arrest while simultaneously promoting ROS production. This generation of ROS is closely associated with cell cycle regulation and the induction of apoptosis. For example, 125I seed promoted the apoptosis of Cholangiocarcinoma cells and induced the activation of the ROS/p53 pathway in a dose-dependent manner. The mechanism may involve the activation of p53 and its downstream apoptotic pathway by upregulating the level of ROS in cells [42]; Walsuronoid B caused G2/M phase arrest and induced mitochondrial and lysosomal apoptosis through the ROS/p53 signaling pathway in human liver cancer cells [19]. This study found that ITSN can significantly induce ROS production in Hep3B cells, upregulate the expression levels of p53 and p21, downregulate the expression level of Cyclin D1 and CDK4, and promote the activation of the apoptosis pathway.

The MAPK and PI3K/AKT pathways are the core regulatory pathways for cell survival and proliferation. Both pathways regulate cell cycle progression and inhibit apoptosis through phosphorylation cascades of downstream signaling molecules. Their abnormal activation is often closely related to abnormal cell proliferation and enhanced anti-apoptotic ability [30,31]. In this study, the inhibitory effect of ITSN on these two pathways can directly relieve the negative regulation of these two pathways on apoptosis and disrupt the normal proliferation signal transmission of cells, creating conditions for the occurrence of apoptosis.

The activation of the apoptosis pathway is the core link of apoptosis. Together, the Bcl-2 and caspase family proteins constitute the mitochondria-mediated endogenous apoptotic pathway. Among these, Bcl-2 acts as a key anti-apoptotic protein, while Bax is a major pro-apoptotic protein. The expression ratio of these two proteins directly determines the cellular apoptotic tendency: the decrease in the Bcl-2/Bax ratio enhances apoptosis, and the increase in the ratio inhibits apoptosis [43]. In this study, Western blot experiments confirmed that ITSN can significantly upregulate the expression of pro-apoptotic protein cleaved-caspase-3 and downregulate the expression of anti-apoptotic protein Bcl-2, and decreased the ratio of Bcl-2/Bax, indicating that ITSN can activate endogenous apoptosis pathway and ultimately induce apoptosis.

Based on the marked inhibitory effect of ITSN on Hep3B cell proliferation, its impact on Hep3B xenograft tumors was assessed in a sterile environment utilizing immunocompromised nude mice. The outcomes revealed that i.g. administration of ITSN led to a substantial reduction in the growth of Hep3B tumor xenografts, and there were no significant changes observed in the body weight. Particularly, ITSN had showed obvious hepatotoxicity in mice. In contrast, several classic natural compounds such as camptothecin, employed in clinical settings with distinctive anticancer properties, are restricted due to associated side effects and toxicity. In addition, the expression trends of the three proteins detected by immunohistochemistry were consistent with the Western blot results in cells. Consequently, ITSN has demonstrated the ability to inhibit tumor progression in vitro and in vivo, showing promising potential for the treatment and prevention of hepatocellular carcinoma. These findings provide more evidence for the use of limonoids as drugs for the treatment of various diseases.

## 5. Conclusions

Accumulating studies have confirmed that naturally occurring limonoids exhibit prominent anti-tumor activity [13]. However, most of the relevant studies are still limited to preliminary activity screening at the in vitro cellular level, and the in-depth molecular mechanisms underlying the anti-tumor effects of limonoids remain to be further clarified. Accordingly, the present study conducted an in-depth investigation on the anti-HCC mechanism of ITSN isolated from *Melia azedarach* bark. In summary, our results indicate that ITSN induces intracellular ROS generation to trigger oxidative stress, inhibits the activation of the MAPK and PI3K/AKT pathways, and then activates the p53 pathway to cause abnormal cell cycle arrest, initiating the mitochondrial-mediated endogenous apoptosis pathway, thereby ultimately inducing HCC cell apoptosis and exerting anti-HCC effects. These findings demonstrate that ITSN is a promising candidate for the therapeutic treatment of liver cancer.

## Data Availability

The original contributions presented in this study are included in the article/Supplementary Materials. Further inquiries can be directed to the corresponding author. Raw RNA-Seq data accession number: PRJNA1445691.

## Supplementary Materials

The following supporting information can be downloaded at: https://www.mdpi.com/article/10.3390/antiox15050562/s1, S1: The detailed information on the main apparatus, reagents, medicinal materials, and some methods used in this study; S2: The original unedited Western blot results; S3: NMR spectra, IR spectra, and MS of the new compounds and the NMR data of all compounds.
