# Supplementary material for: Anti-Hepatocarcinoma Activity and Mechanism of Isosendanin and Its Novel Structural Analogues Isolated from the Bark of Melia azedarach L.: In Vitro and In Vivo Studies
Source: Antioxidants (Basel). 2026 Apr 29;15(5):562. doi: 10.3390/antiox15050562 (PMC13203457; doi:10.3390/antiox15050562)
Supplement: Supplementary file 1 [file antioxidants-15-00562-s001.zip › Supplementary Material S1.pdf]

Supplementary Material S1

Anti-Hepatocarcinoma Activity and Mechanism of Isosendanin and Its Novel Structural Analogues Isolated from the Bark of *Melia azedarach* L.: In Vitro and In Vivo Studies

1. Materials and methods

1.1 Main Apparatus

IR spectra were recorded on a Spectrum TWO Fourier Transform Infrared Spectrometer (PerkinElmer, USA). NMR spectra were measured on a Bruker Avance III 500 MHz Nuclear Magnetic Resonance System (Bruker, Germany). Mass spectrum data were acquired with a SCIEX X500R High-Resolution Liquid Chromatography-Mass Spectrometer (AB SCIEX, Singapore). HPLC separation was conducted using an Agilent 1260 Infinity II preparative high-performance liquid chromatograph (Agilent, USA) and a COSMOSIL Packed Column ($5C_{18}$-MS-II, 10 mm × 250 mm, 5 μm). Silica gel (100-200, 200-300 mesh) was from Qingdao Marine Chemical Plant, Qingdao, China), MCI gel (CHP20/P120, 75-150 μm, Tokyo, Japan) and Sephadex LH-20 (GE Healthcare, Tokyo, Japan) were used for column chromatography. Preparative TLC was carried out with GF 254 plates (Qingdao Marine Chemical Factory, Qingdao, China). A carbon dioxide incubator was used for cell culture (Thermo Fisher Scientific, USA), and a microplate reader was employed for the determination of OD values (Thermo Fisher Scientific, USA). DMI8 inverted fluorescence microscope for staining analysis (Leica, Germany). Cell apoptosis was analyzed by BD LSRFortessa flow cytometry (Becton, Dickinson and Company, USA).

1.2 Main Reagents

Acetonitrile and Methanol (chromatographically pure, Shanghai Aladdin Biochemical Technology Co., Ltd., China); all other reagents used were of domestic analytical grade with a purity of ⩾ 99.5%. Fetal Bovine Serum (FBS), DMEM high-glucose medium, and penicillin-streptomycin double-antibody solution (Pen Strep, 100×) were purchased from Gibco (USA); 3-(4,5-Dimethylthiazol-2-yl)-2,5-diphenyltetrazolium bromide (MTT), Hoechst 33342 Stain Solution(ready-to-use) (Cat：C0030), and RIPA Buffer(high) (Cat：R0010) were purchased from Beijing Solarbio Science & Technology Co., Ltd. (China). TRIzol reagent (batch number: 15596026CN) was purchased from invitrogen by Thermo Fisher Scientific; Annexin V-FITC/PI Apoptosis Detection Kit (Cat: KGA1102-50) was purchased from KeyGEN BioTECH; Enhanced

BCA Protein Assay Kit (Cat: P0009) was purchased from Beyotime;

1.3. Plant Material

Melia azedarach L.was purchased from Guilin, Guangxi in May 2024. It was identified as the barks of *Melia azedarach* L. by the deputy chief pharmacist Zhong Xiaoqing of Guilin Sanjin Co., Ltd. Plant samples (202405MA) were stored in the institute of traditional Chinese and Zhuang-Yao ethnic medicine, Guangxi University of Chinese Medicine, Nanning, China.

1.4 Detailed information on primary and secondary antibodies

Antibody	Source	Catalogue number	Concentration	Antibody company
Primary Antibody				
ERK	Rabbit	ER131218	1:4000	huabio
P-ERK	Rabbit	5726T	1:1000	Cell Signaling Technology
P-JNK	Rabbit	T40074	1:1000	Abmart
JUN	Rabbit	24909-1-AP	1:4000	proteintech
MMP9	Rabbit	10375-2-AP	1:2000	proteintech
TLR4	Mouse	66350-1-Ig	1:4000	proteintech
MyD88	Rabbit	AF2116	1:1000	Beyotime
TNF-α	Rabbit	17590-1-AP	1:2000	proteintech
IL-6	Mouse	66146-1-Ig	1:3000	proteintech
PI3K	Rabbit	HA722522	1:1000	huabio
p-PI3K	Rabbit	HA721672	1:1000	huabio
AKT1	Rabbit	AF0836	1:1000	Affinity
p-AKT1	Rabbit	AF0832	1:1000	Affinity
GSK3β	Rabbit	22104-1-AP	1:5000	proteintech
p-GSK3β	Mouse	67558-1-Ig	1:4000	proteintech
p53	Rabbit	10442-1-AP	1:5000	proteintech
p21	Rabbit	10355-1-AP	1:2000	proteintech
Cyclin D1	Rabbit	26939-1-AP	1:5000	proteintech
CDK4	Rabbit	11026-1-AP	1:4000	proteintech
Bax	Rabbit	50599-2-Ig	1:100000	proteintech
Bcl-2	Rabbit	26593-1-AP	1:2000	proteintech
Cleaved-caspase 3	Rabbit	25128-1-AP	1:2000	proteintech
Secondary Antibody				
Anti-Rabbit IgG H&L	Goat	bs-0295G	1:5000/1:2000	Bioss
Anti-Mouse IgG H&L	Goat	bs-0296G	1:5000/1:2000	Bioss

1.5 Immunohistochemical Analysis

1) Tissue section preparation

Hepatocellular carcinoma tissues were isolated from nude mice. After routine gradient dehydration, clearing, wax impregnation and embedding, paraffin tissue blocks were prepared. Continuous sections at a thickness of 4 μm were cut using a microtome. The sections were flattened and mounted on adhesive glass slides, then baked in a constant-temperature oven at 60 °C for 2 h to ensure firm attachment for subsequent use.

2) Dewaxing and rehydration

Paraffin sections were dewaxed and rehydrated in sequence: xylene three times (10 min, 10 min, 5 min), absolute ethanol twice for 5 min each, 95% ethanol for 5 min, 85% ethanol for 5 min, and finally rinsed with distilled water for 5 min.

3) Antigen retrieval

The sections were immersed in 0.01 mol/L citrate antigen retrieval buffer (pH = 6.0). Antigen retrieval was performed using a high-temperature and high-pressure method. The solution was heated to boiling and maintained for 20 min, then cooled naturally to room temperature. The sections were rinsed three times with PBS for 5 min each to remove residual buffer.

4) Inactivation of endogenous peroxidase

3% hydrogen peroxide solution was added dropwise and incubated at room temperature for 15 min in the dark to block endogenous peroxidase activity and prevent non-specific staining. After incubation, the sections were washed three times with PBS for 5 min each.

5) Blocking

After slightly drying the sections, a hydrophobic pen was used to draw circles around the tissues to avoid liquid loss. Appropriate goat serum blocking solution was added dropwise within the circled area, and the sections were blocked at room temperature for 20 min to block non-specific binding sites and reduce background staining.

6) Primary antibody incubation

The blocking solution was gently discarded. The prepared primary antibody solution was evenly added to the sections and incubated overnight at 4 °C. Afterwards, the sections were rinsed gently with PBS three times for 5 min each.

7) Secondary antibody incubation

The corresponding species-specific secondary antibody was added dropwise within the hydrophobic circle. The sections were incubated at room temperature for 30 min away from light, followed by gentle PBS washing three times for 5 min each.

8) DAB color development

Freshly prepared DAB working solution was applied to the sections. The color reaction was observed under a light microscope with the reaction time controlled within 3–10 min. Once specific brown-yellow staining appeared with a clear background, the reaction was immediately terminated by rinsing with distilled water.

9) Counterstaining

Sections were counterstained with hematoxylin for 30 s, followed by rinsing with distilled water for 3 min.

10) Dehydration and clearing

Sections were dehydrated sequentially in 85% ethanol for 2 min, 95% ethanol for 2 min, absolute ethanol twice for 5 min each, and cleared with xylene for 1–2 min.

11) Mounting and microscopic examination

After clearing and air drying, neutral balsam was applied, and coverslips were carefully mounted. After complete drying, histological staining was observed under an optical microscope. Typical visual fields were captured, and image analysis software was used for quantitative detection of positive expression area and optical density.
