# Supplementary material for: Anti-Hepatocarcinoma Activity and Mechanism of Isosendanin and Its Novel Structural Analogues Isolated from the Bark of Melia azedarach L.: In Vitro and In Vivo Studies
Source: Antioxidants (Basel). 2026 Apr 29;15(5):562. doi: 10.3390/antiox15050562 (PMC13203457; doi:10.3390/antiox15050562)
Supplement: Supplementary file 1 [file antioxidants-15-00562-s001.zip › Supplementary Material S2.pdf]

Supplementary Material S2

Anti-Hepatocarcinoma Activity and Mechanism of Isosendanin and Its Novel Structural Analogues Isolated from the Bark of *Melia azedarach* L.: In Vitro and In Vivo Studies

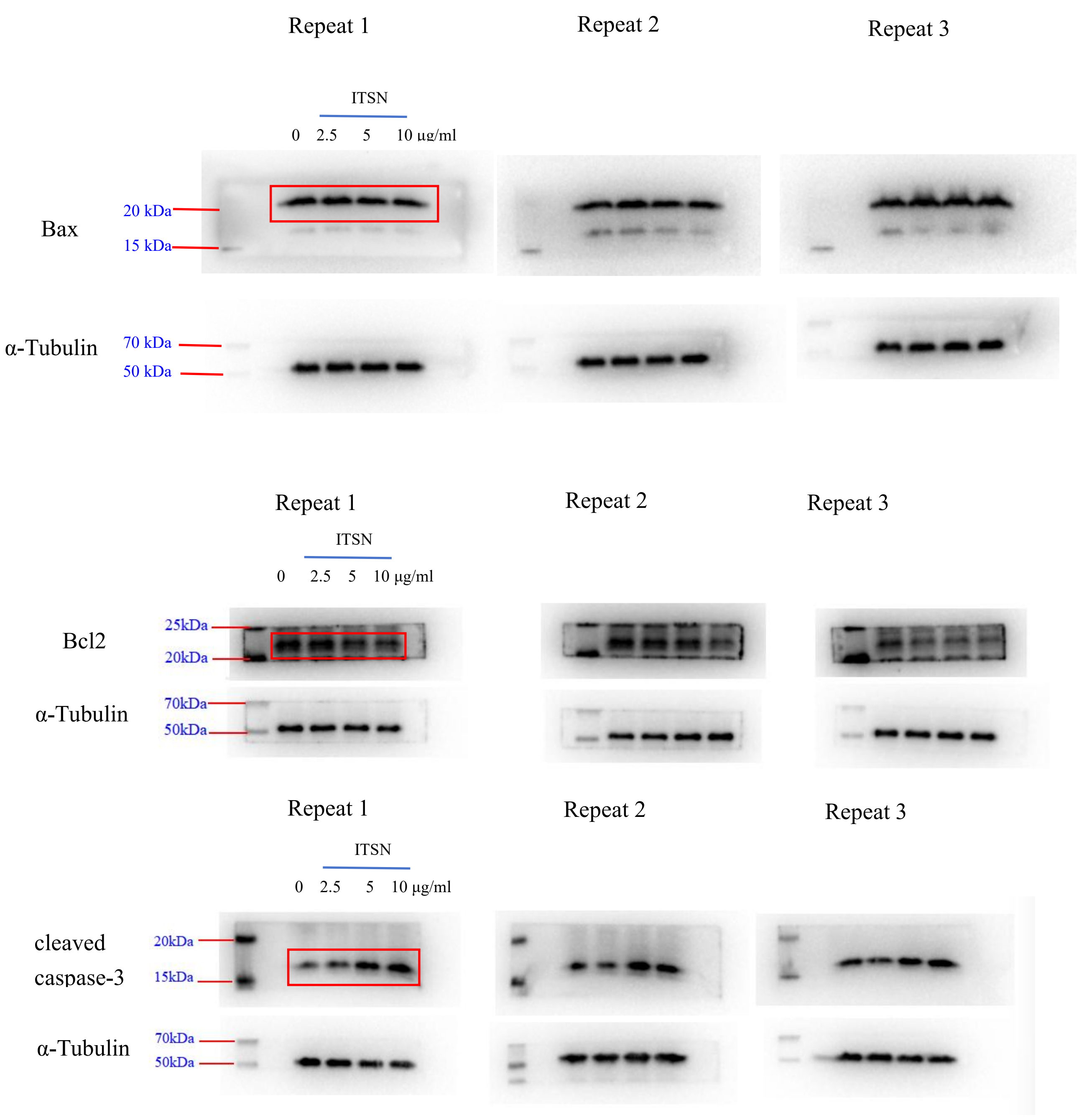

Supplementary Figure S1. Original unprocessed X-ray film of western blot corresponding to Figure 6C. The blot displays Bax, Bcl2, cleaved caspase-3, and α-Tubulin bands, which have three repeated experiments.

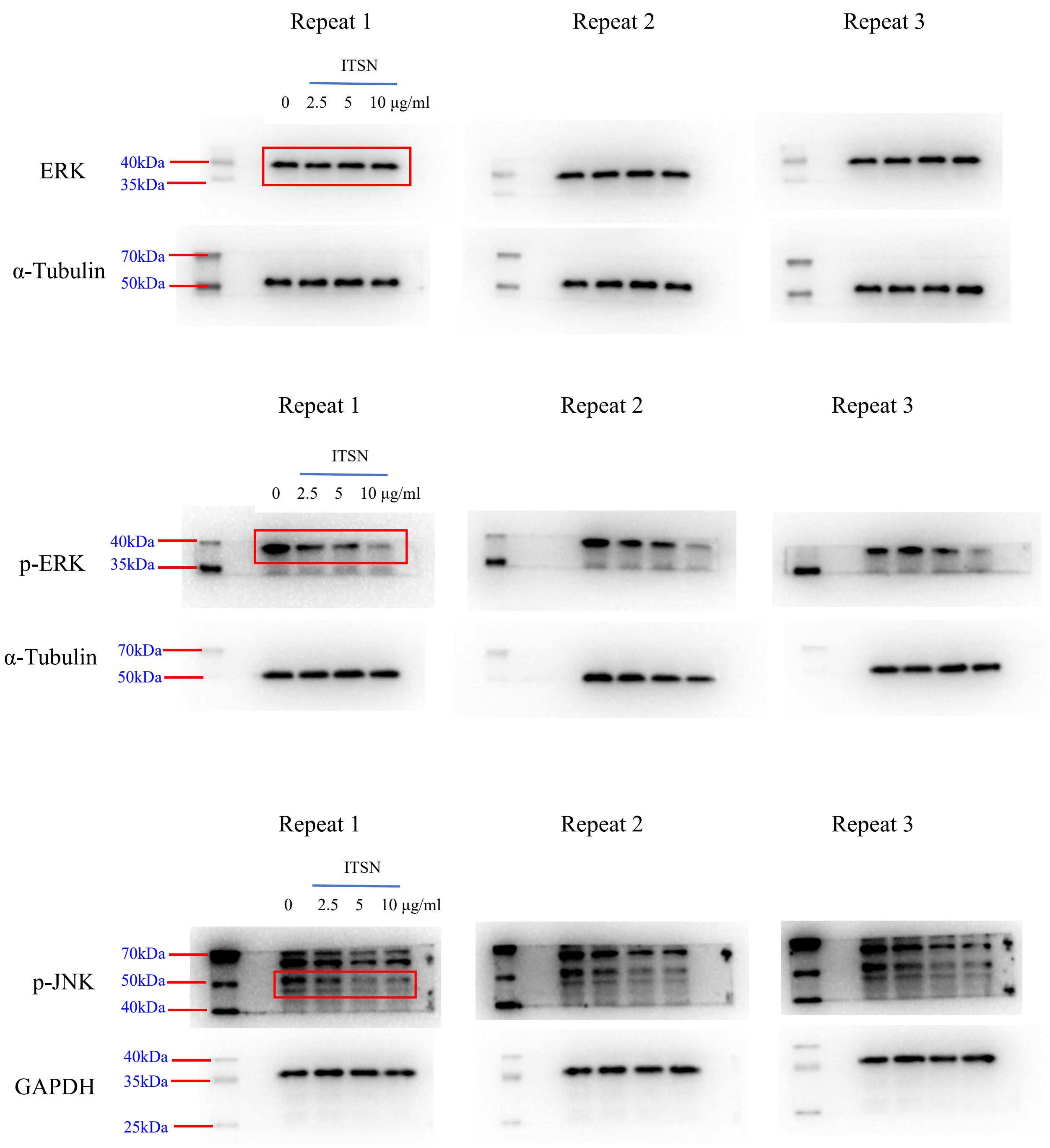

Supplementary Figure S1. Original unprocessed X-ray film of western blot corresponding to Figure 7. The blot displays ERK, p-ERK, p-JNK, α-Tubulin and GAPDH bands, which have three repeated experiments.

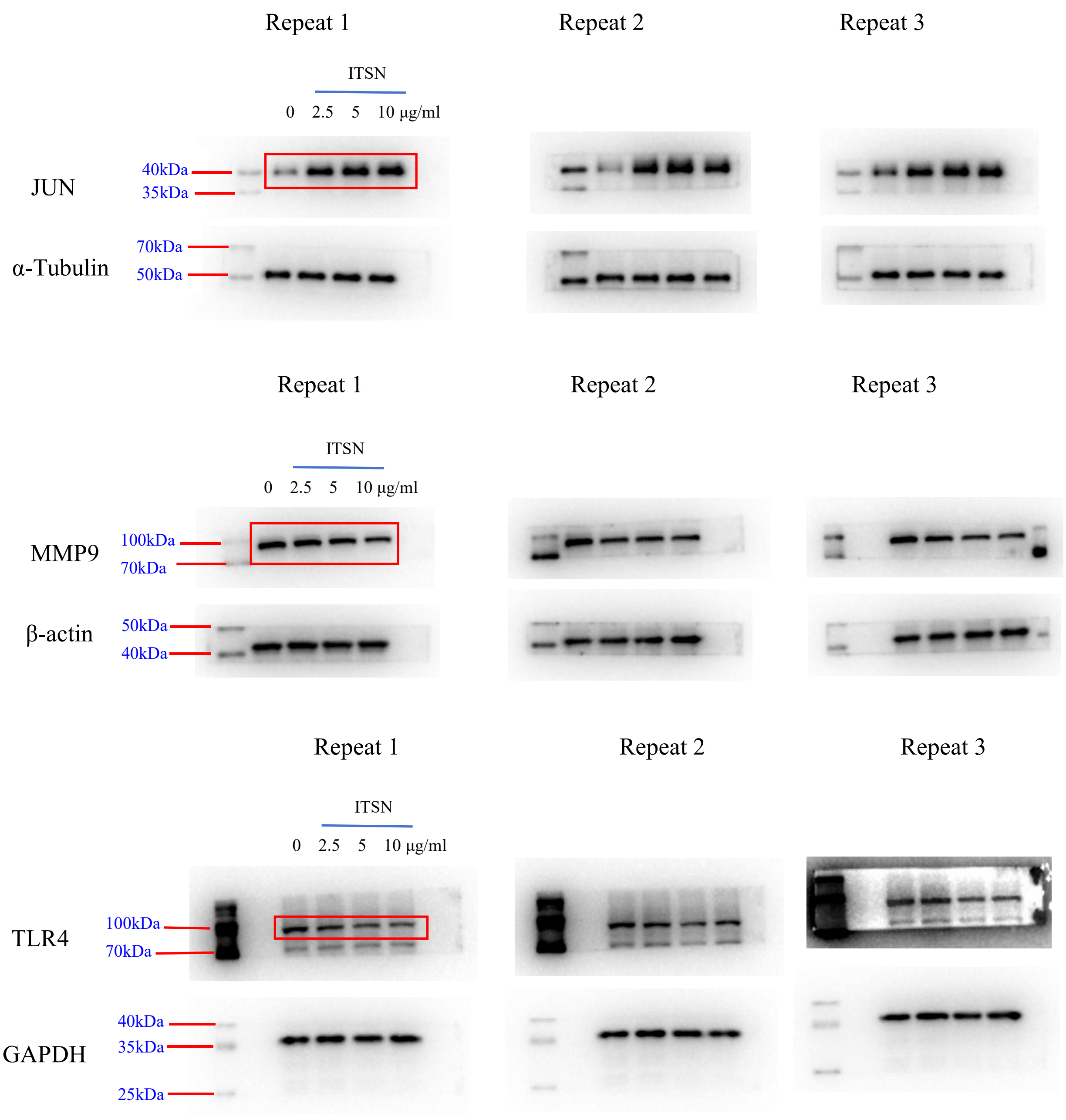

Supplementary Figure S1. Original unprocessed X-ray film of western blot corresponding to Figure 7. The blot displays JUN, MMP9, TLR4, α-Tubulin, β-actin and GAPDH bands, which have three repeated experiments.

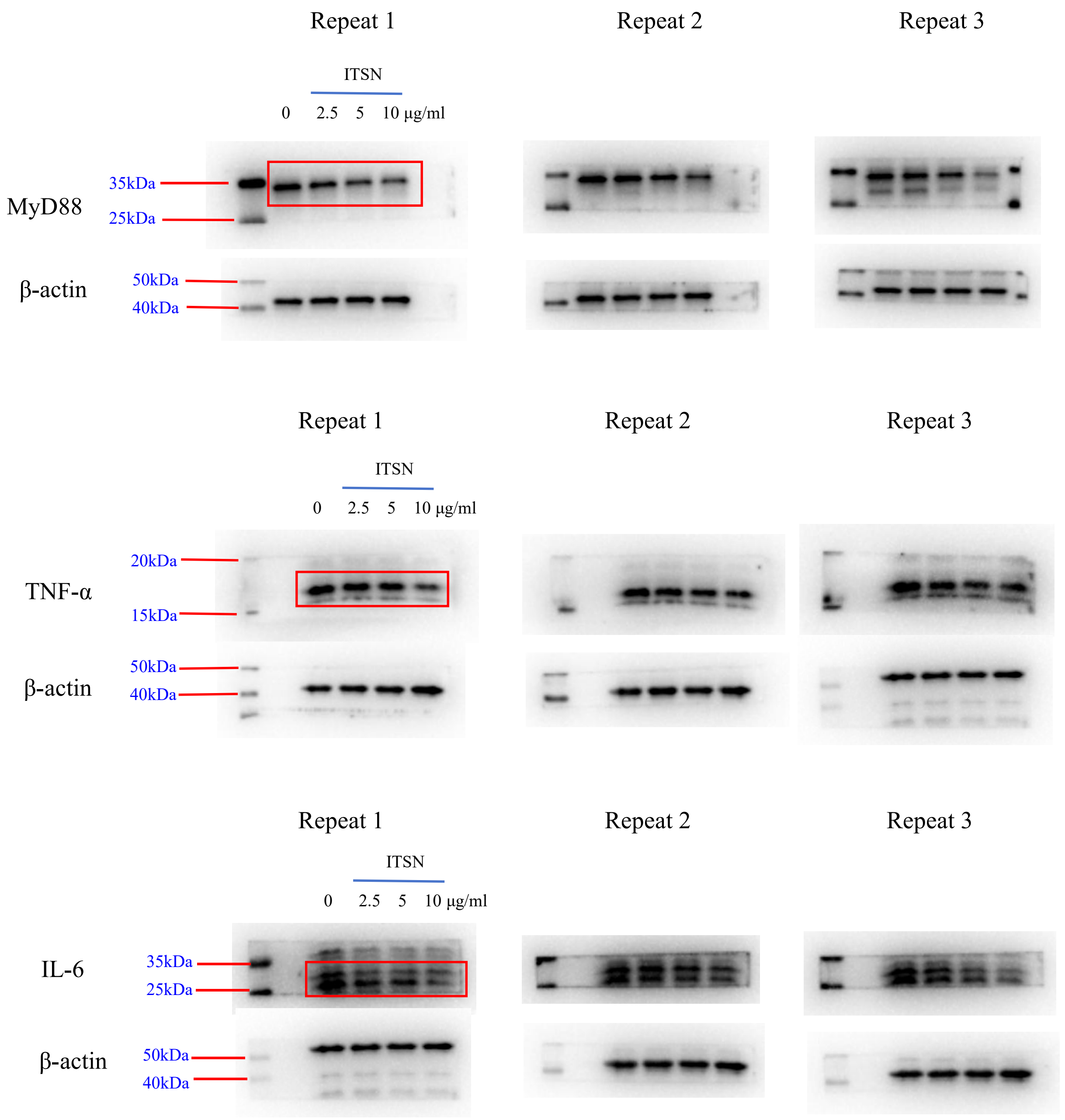

Supplementary Figure S1. Original unprocessed X-ray film of western blot corresponding to Figure 7. The blot displays MyD88, TNF-α, IL-6, and β-actin bands, which have three repeated experiments.

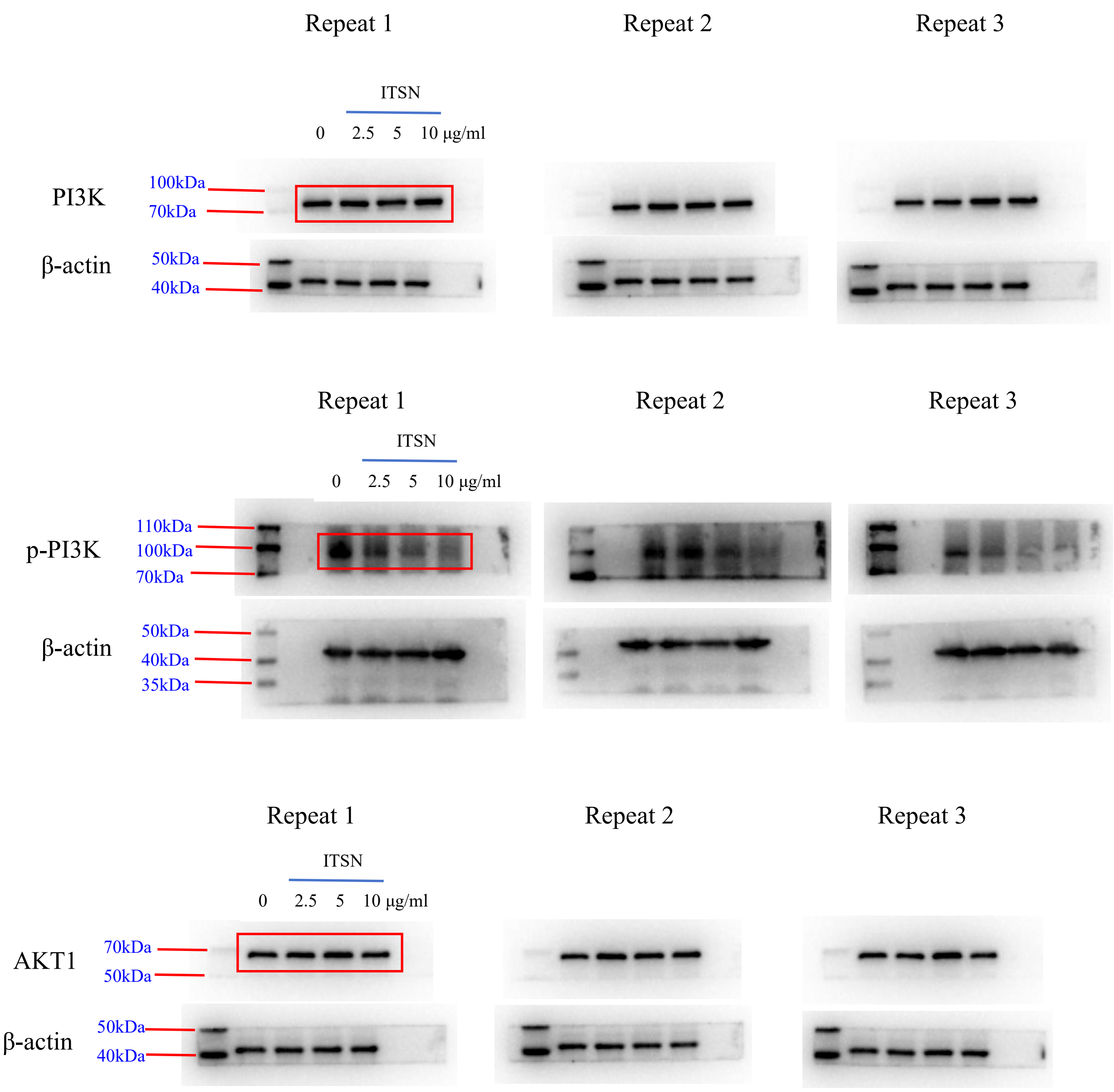

Supplementary Figure S1. Original unprocessed X-ray film of western blot corresponding to Figure 7. The blot displays PI3K, p-PI3K, AKT1, and β-actin bands, which have three repeated experiments.

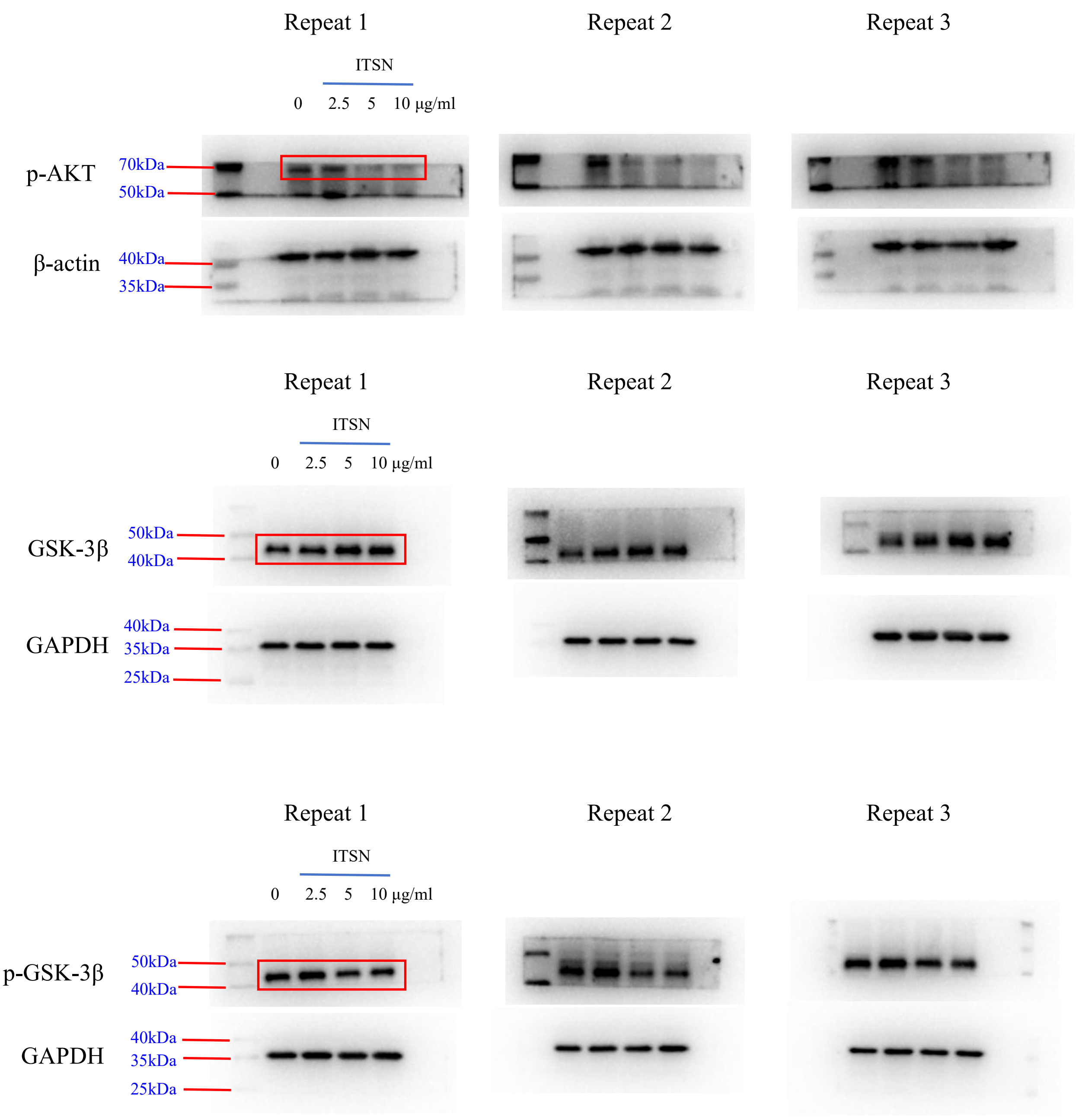

Supplementary Figure S1. Original unprocessed X-ray film of western blot corresponding to Figure 7. The blot displays p-AKT1, GSK3-β, p-GSK3-β, β-actin and GAPDH bands, which have three repeated experiments.

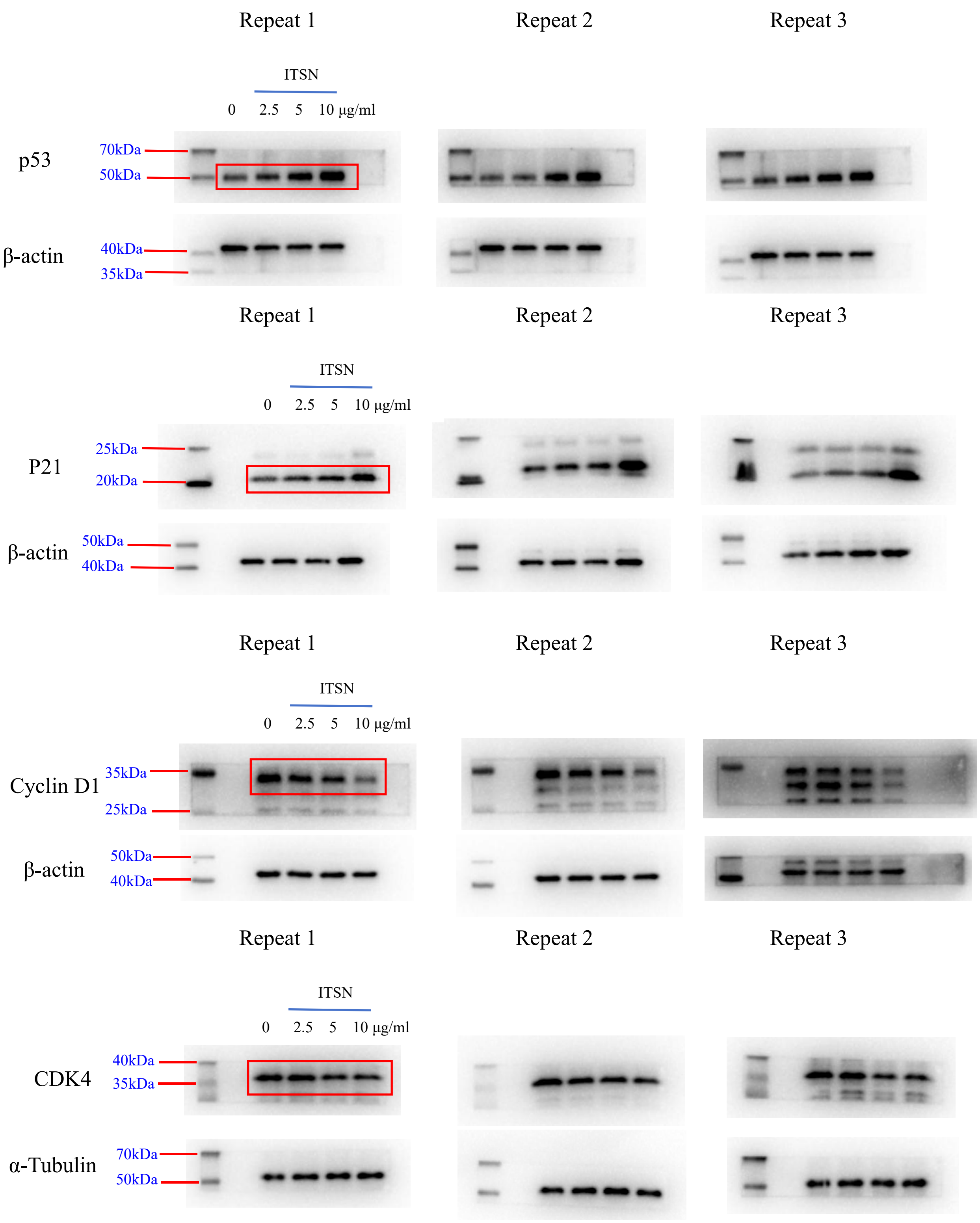

Supplementary Figure S1. Original unprocessed X-ray film of western blot corresponding to Figure 8. The blot displays p53, p21, Cyclin D1, CDK4, β-actin and α-Tubulin bands, which have three repeated experiments.
