# Supplementary material for: Anti-Hepatocarcinoma Activity and Mechanism of Isosendanin and Its Novel Structural Analogues Isolated from the Bark of Melia azedarach L.: In Vitro and In Vivo Studies
Source: Antioxidants (Basel). 2026 Apr 29;15(5):562. doi: 10.3390/antiox15050562 (PMC13203457; doi:10.3390/antiox15050562)

Supplementary Material S3

Anti-Hepatocarcinoma Activity and Mechanism of Isosendanin and Its Novel Structural Analogues Isolated from the Bark of *Melia azedarach* L.: In Vitro and In Vivo Studies

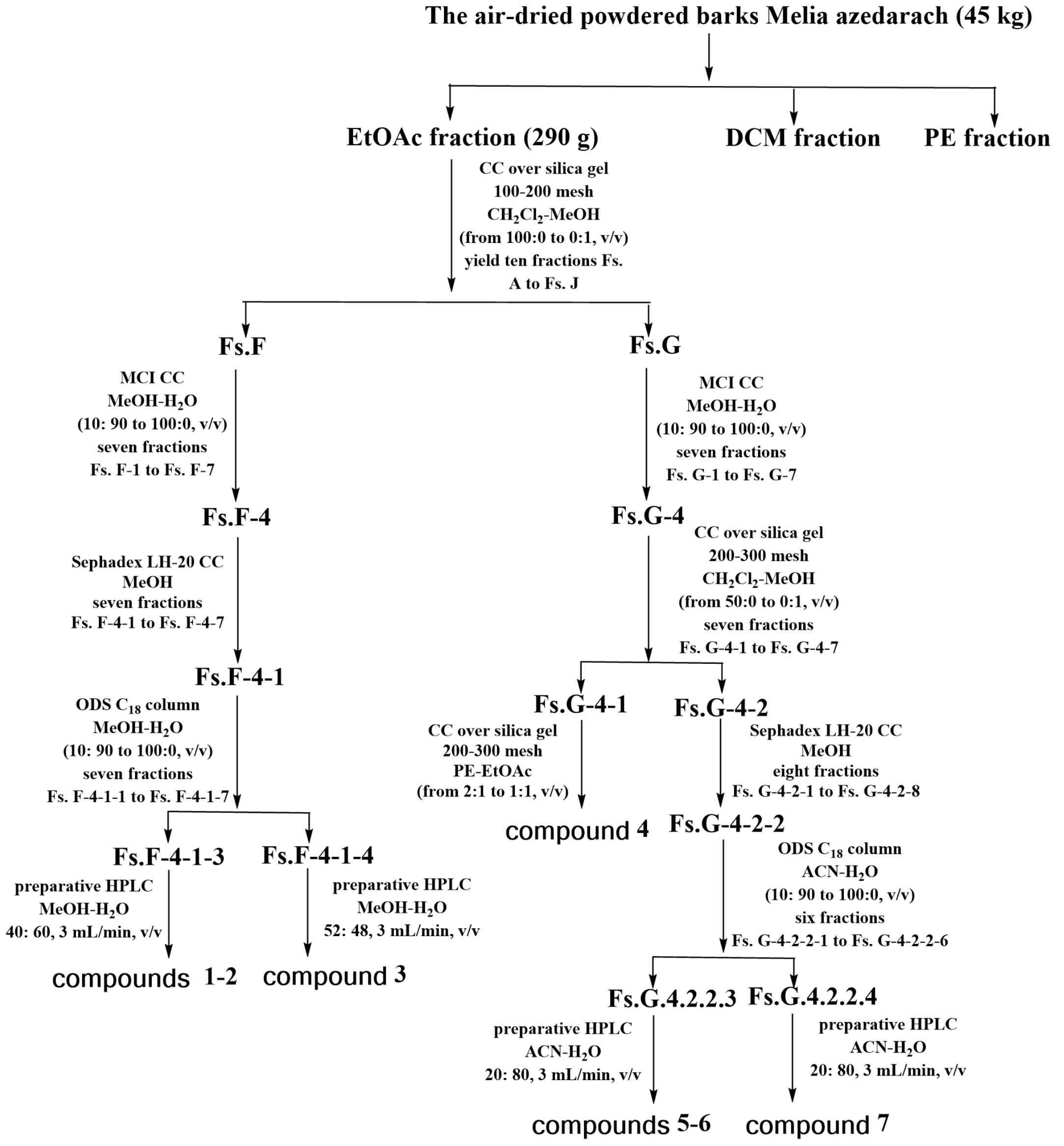

Figure S1 Separation Flow Chart

1. NMR analysis of compounds 1-7:

Table S1 ^{1}H and ^{13}C NMR data of compounds **1** and **3** (δ in ppm)

Position	**1**		**3**	
	δ_H	δ_C	δ_H	δ_C
1	4.51 (1H, br s)	73.4	3.95 (1H, m)	71.0
2α	1.82 (1H, m)	38.6	2.09 (2H, m)	31.9
2β	2.74 (1H, m)		-	
3	3.45 (1H, br s)	76.2	5.19 (1H, br s)	74.6
4	-	42.6	-	46.5
5	2.73 (1H, m)	25.6	2.55 (1H, m)	28.3
6α	1.67 (1H, m)	26.4	1.67 (1H, m)	25.8
6β	2.43 (1H, m)		1.97 (1H, m)	
7	4.02 (1H, br s)	70.6	3.94 (1H, m)	69.2
8	-	43.1	-	45.0
9	2.64 (1H, m)	43.5	3.48 (1H, d, J = 8.5 Hz)	53.1
10	-	43.6	-	40.2
11	4.43 (1H, dd, J = 11.7, 1.6 Hz)	73.1	-	210.6
12α	-	214.3	2.32 (1H, d, J = 17.5 Hz)	50.3
12β	-		2.72 (1H, d, J = 17.6 Hz)	
13	-	54.0	-	43.7
14	3.24 (1H, s)	64.3	3.15 (1H, s)	63.1
15	-	218.8	-	219.5
16α	2.54 (1H, m)	43.4	2.60 (2H, d, J = 10.3 Hz)	43.5
16β	2.69 (1H, m)		-	
17	3.72 (1H, m)	39.1	3.37 (1H, t, J = 10.6 Hz)	42.0
18	1.12 (3H, s)	23.1	1.04 (3H, s)	28.5
19α	3.67 (1H, d, J = 11.5 Hz)	60.4	4.68 (1H, d, J = 14.2 Hz)	72.5
19β	4.30 (1H, d, J = 11.5 Hz)		4.78 (1H, d, J = 14.1 Hz)	
20	-	124.4	-	121.8
21	7.38 (1H, s)	142.1	7.43 (1H, s)	143.7
22	6.42 (1H, s)	112.3	6.31 (1H, s)	110.4
23	7.42 (1H, s)	143.9	7.32 (1H, s)	140.4
28	0.97 (3H, s)	19.3	1.21 (3H, s)	19.7
29	4.11 (1H, s)	105.0	-	172.7
30	1.11 (3H, s)	19.2	1.11 (3H, s)	20.1
29-OCH_3	3.33 (3H, s)	55.7	-	-
3-$OCO\underline{CH_3}$	-	-	2.14 (3H, s)	21.2
3-$O\underline{C}OCH_3$	-	-	-	169.0

12-hydroxyamoorastatone (**2**): ^{1}H NMR (500 MHz, DMSO-d_6) δ: 7.41 (1H, s, H-23), 7.33 (1H, s, H-21), 6.34 (1H, s, H-22), 5.17 (1H, d, J = 4.0 Hz, H-3), 4.83 (1H, s, H-29), 4.54 (1H, d, J = 4.1 Hz, H-1), 4.22 (1H, s, H-12), 3.65 (1H, s, H-9), 3.33 (1H, s, H-14), 2.04 (3H, s, 3-OAc), 1.10 (3H, s,

H-30), 0.88 (3H, s, H-18), 0.83 (3H, s, H-28); ^{13}C NMR (125 MHz, DMSO-d_6) δ: 71.3 (C-1), 37.2 (C-2), 74.8 (C-3), 41.0 (C-4), 29.3 (C-5), 24.0 (C-6), 70.5 (C-7), 42.7 (C-8), 49.0 (C-9), 43.6 (C-10), 214.0 (C-11), 78.8 (C-12), 47.5 (C-13), 59.4 (C-14), 220.6 (C-15), 46.3 (C-16), 39.7 (C-17), 22.1 (C-18), 65.0 (C-19), 126.3 (C-20), 141.5 (C-21), 111.9 (C-22), 144.2 (C-23), 19.9 (C-28), 97.2 (C-29), 22.2 (C-30), 21.3 (3-OAc), 172.8 (3-OAc).

Meliarachin B (**4**): ^{1}H NMR (500 MHz, DMSO-d_6) δ: 7.55 (1H, s, H-23), 7.36 (1H, s, H-21), 6.13 (1H, s, H-22), 5.27 (1H, s, H-12), 4.97 (1H, s, H-3), 4.73 (1H, d, J = 13.8 Hz, H-19β), 4.57 (1H, s, H-9), 4.37 (1H, d, J = 13.9 Hz, H-19α), 3.96 (1H, br s, H-1), 2.03 (3H, s, 3-OAc), 1.94 (3H, s, 3-OAc), 1.03 (3H, s, H-30), 1.25 (3H, s, H-18), 1.00 (3H, s, H-28); ^{13}C NMR (125 MHz, DMSO-d_6) δ: 68.4 (C-1), 31.5 (C-2), 73.1 (C-3), 45.9 (C-4), 27.1 (C-5), 27.7 (C-6), 67.9 (C-7), 42.3 (C-8), 48.6 (C-9), 38.7 (C-10), 207.8 (C-11), 77.2 (C-12), 45.1 (C-13), 71.9 (C-14), 58.1 (C-15), 33.2 (C-16), 38.1 (C-17), 14.8 (C-18), 72.7 (C-19), 122.6 (C-20), 140.8 (C-21), 111.9 (C-22), 142.8 (C-23), 19.9 (C-28), 173.4 (C-29), 21.2 (C-30), 20.5 (3-OAc), 170.0 (3-OAc), 21.5 (12-OAc), 169.7 (12-OAc).

Meliarachin I (**5**): ^{1}H NMR (500 MHz, DMSO-d_6) δ: 7.57 (1H, s, H-23), 7.44 (1H, s, H-21), 6.32 (1H, s, H-22), 4.01 (1H, d, J = 11.8 Hz, H-19β), 3.83 (1H, d, J = 11.8 Hz, H-19α), 3.61 (1H, s, H-9), 3.36 (1H, m, H-3), 3.26 (1H, s, H-14), 3.21 (3H, s, 29-OCH_3), 0.96 (3H, s, H-30), 0.81 (3H, s, H-29), 0.76 (3H, s, H-18); ^{13}C NMR (125 MHz, DMSO-d_6) δ: 70.9 (C-1), 37.9 (C-2), 73.8 (C-3), 41.0 (C-4), 23.6 (C-5), 25.2 (C-6), 68.4 (C-7), 42.2 (C-8), 47.1 (C-9), 42.0 (C-10), 213.4 (C-11), 76.7 (C-12), 46.3 (C-13), 57.5 (C-14), 218.6 (C-15), 44.7 (C-16), 37.7 (C-17), 21.2 (C-18), 58.0 (C-19), 124.9 (C-20), 140.2 (C-21), 111.2 (C-22), 143.2 (C-23), 18.9 (C-28), 102.8 (C-29), 21.6 (C-30), 55.0 (29-OCH_3).

Isotoosendanin (**6**): ^{1}H NMR (500 MHz, DMSO-d_6) δ: 7.61 (1H, s, H-23), 7.50 (1H, s, H-21), 6.39 (1H, s, H-22), 4.98 (1H, d, J = 4.3 Hz, H-3), 4.89 (1H, s, H-12), 4.72 (2H, d, J = 4.0 Hz, H-29), 4.08 (1H, d, J = 12.3 Hz, H-19β), 3.96 (1H, d, J = 12.3 Hz, H-19α), 3.88 (1H, m, H-1), 3.81 (1H, m, H-7), 2.03 (3H, s, 12-OAc), 1.96 (3H, s, H-18), 1.01 (3H, s, 3-OAc), 0.88 (3H, s, H-30), 0.67 (3H, s, H-28); ^{13}C NMR (125 MHz, DMSO-d_6) δ: 68.9 (C-1), 35.9 (C-2), 72.6 (C-3), 39.3 (C-4), 27.4 (C-5), 22.9 (C-6), 68.1 (C-7), 43.9 (C-8), 46.8 (C-9), 41.4 (C-10), 207.9 (C-11), 79.0 (C-12), 46.3 (C-13), 58.3 (C-14), 216.9 (C-15), 43.7 (C-16), 37.9 (C-17), 21.5 (C-18), 63.3 (C-19), 123.1 (C-20), 140.6

(C-21), 111.1 (C-22), 143.4 (C-23), 95.2 (C-29), 19.3 (C-28), 21.3 (C-30), 20.3 (3-OAc), 20.7 (12-OAc), 170.0 (3-OAc), 170.2 (12-OAc).

Meliarachin F (**7**): ^{1}H NMR (500 MHz, DMSO-d_6) δ: 7.49 (1H, s, H-23), 7.33 (1H, s, H-21), 6.41 (1H, s, H-22), 4.79 (1H, s, H-7), 4.64 (1H, m, H-15), 4.54 (1H, m, H-3), 4.48 (1H, br s, H-1), 4.15 (1H, d, J = 11.5 Hz, H-19β), 3.59 (1H, s, H-12), 3.43 (1H, d, J = 11.7 Hz, H-19α), 1.97 (3H, s, 3-OAc), 1.17 (3H, s, H-28), 0.81 (3H, s, H-18), 0.74 (3H, s, H-30); ^{13}C NMR (125 MHz, DMSO-d_6) δ: 67.8 (C-1), 36.2 (C-2), 76.1 (C-3), 40.0 (C-4), 28.7 (C-5), 23.9 (C-6), 81.7 (C-7), 42.8 (C-8), 51.5 (C-9), 38.2 (C-10), 209.9 (C-11), 79.2 (C-12), 50.0 (C-13), 96.5 (C-14), 74.6 (C-15), 37.3 (C-16), 42.6 (C-17), 14.9 (C-18), 58.1 (C-19), 126.1 (C-20), 139.9 (C-21), 112.8 (C-22), 142.3 (C-23), 17.8 (C-28), 95.4 (C-29), 18.2 (C-30), 170.3 (3-OAc).

2. NMR spectra, IR spectra, MS of the new compounds.

Figure S2 NMR analysis of 11*α*-hydroxy-12-*Oxo*-Meliarachin I (**1**) (A, IR; B, HR-ESI-MS; C, ^{1}H NMR; D, ^{13}C NMR; E, DEPT135° ; F, HSQC; G, HMBC; H, ^{1}H-^{1}H COSY; I, NOESY)

Figure S2A

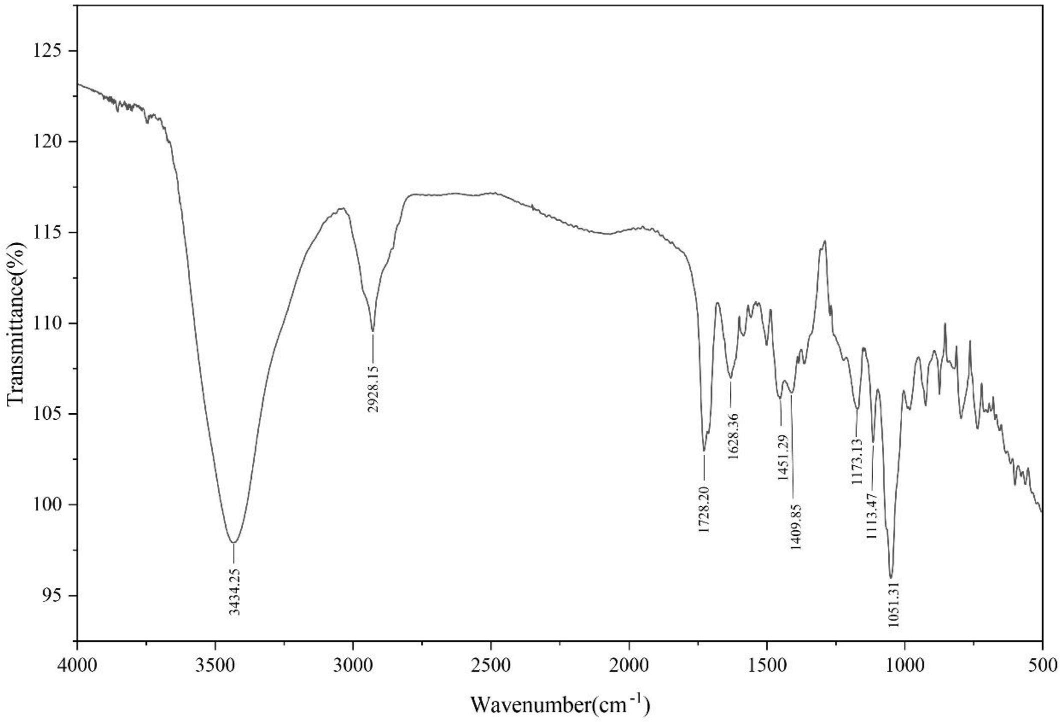

Figure S2B

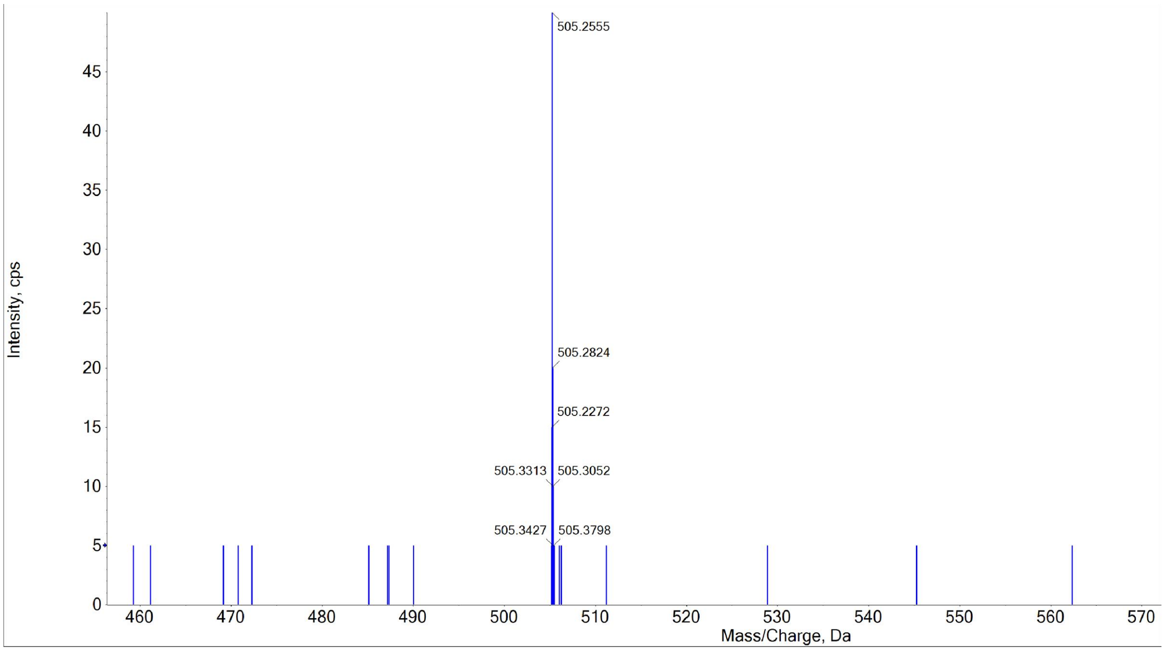

Figure S2C

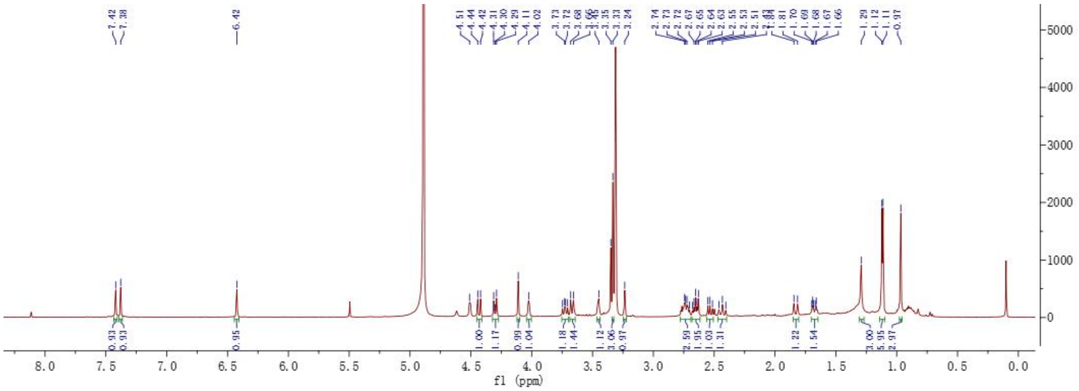

Figure S2D

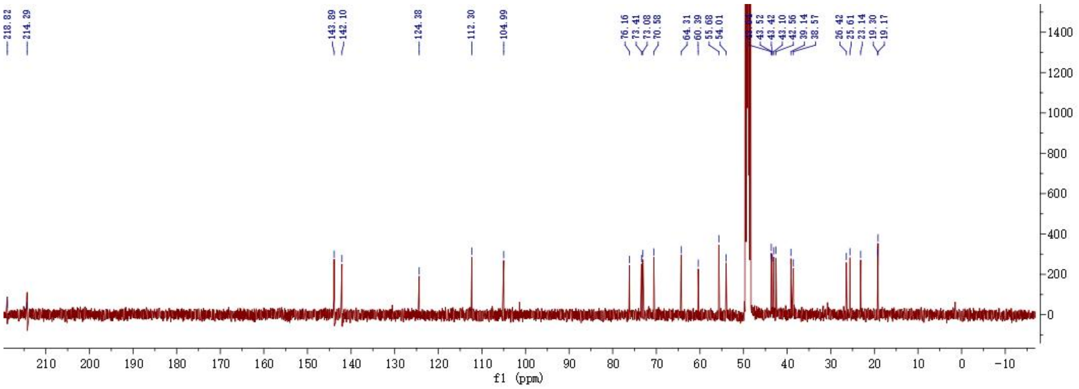

Figure S2E

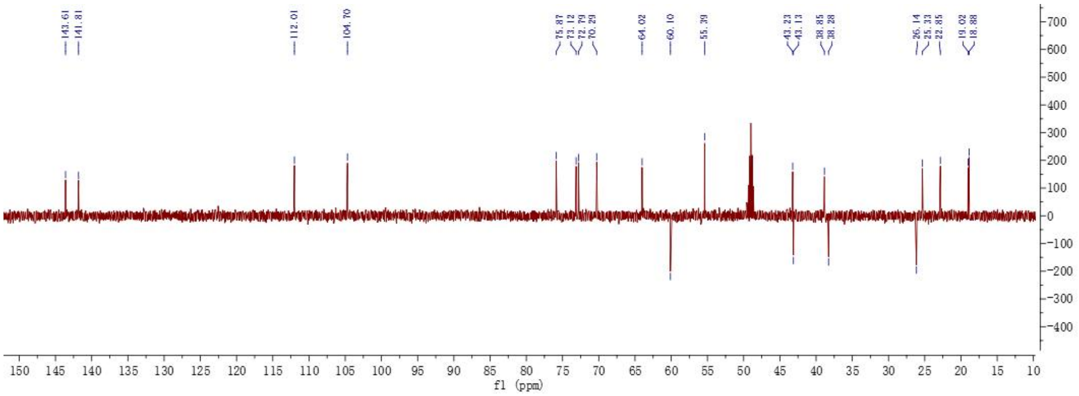

Figure S2F

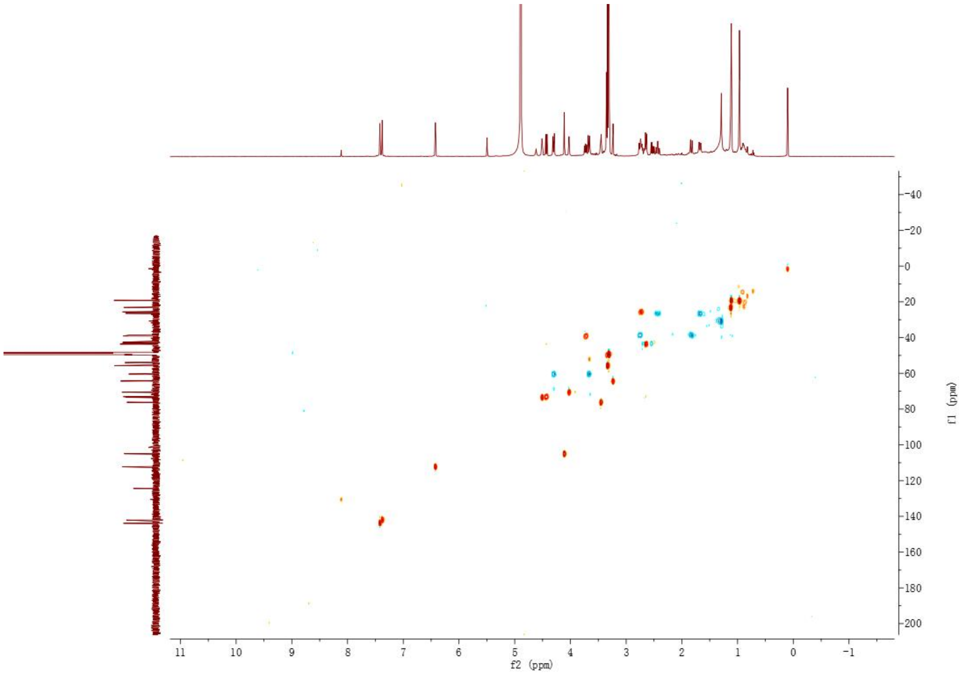

Figure S2G

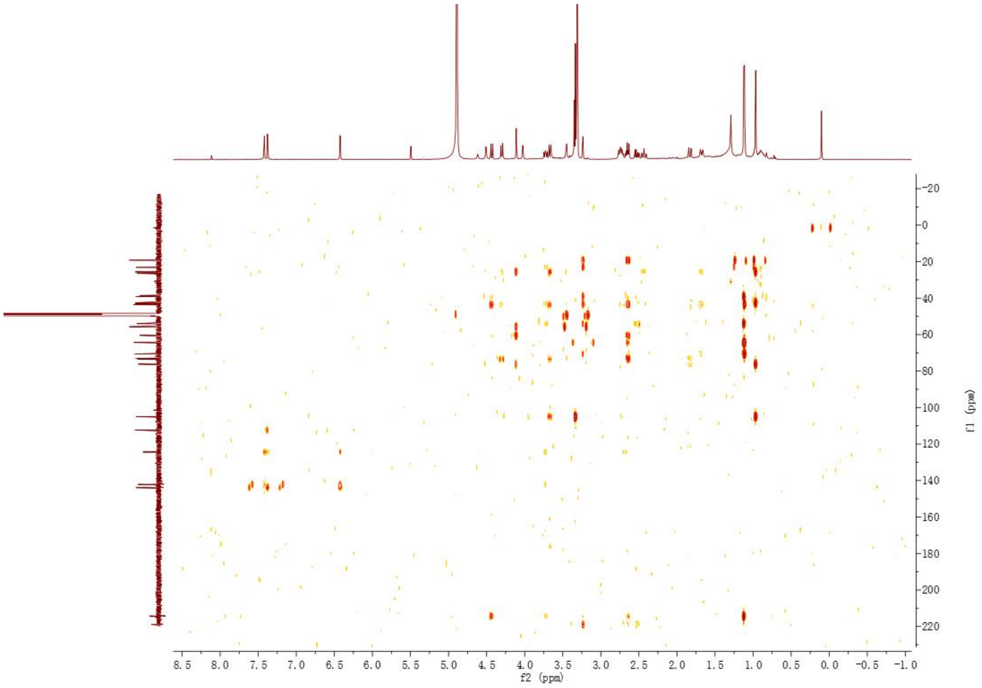

Figure S2H

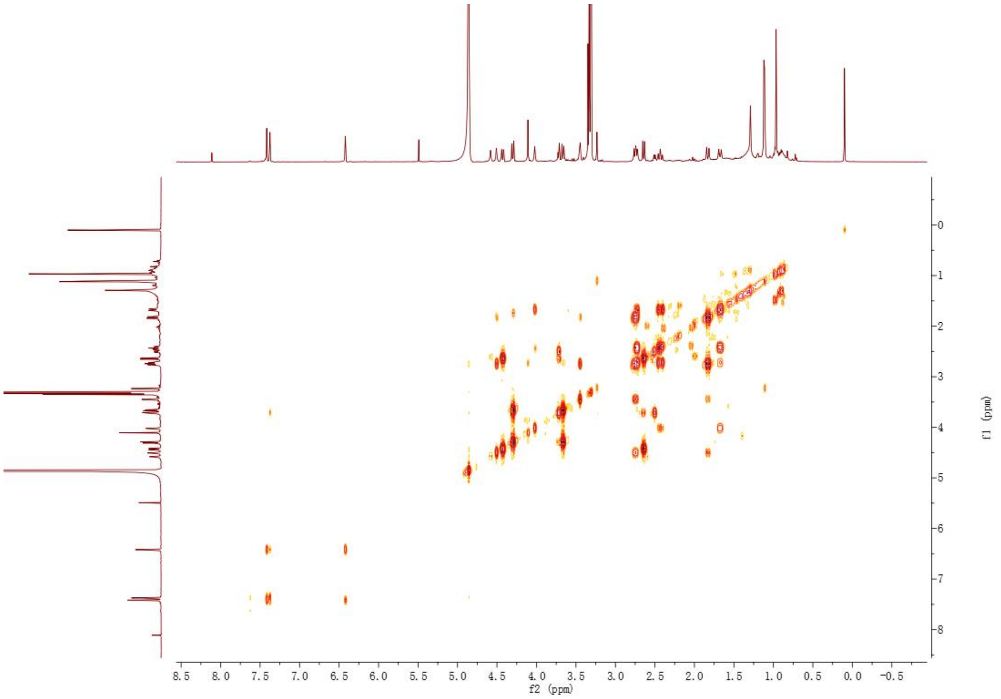

Figure S2I

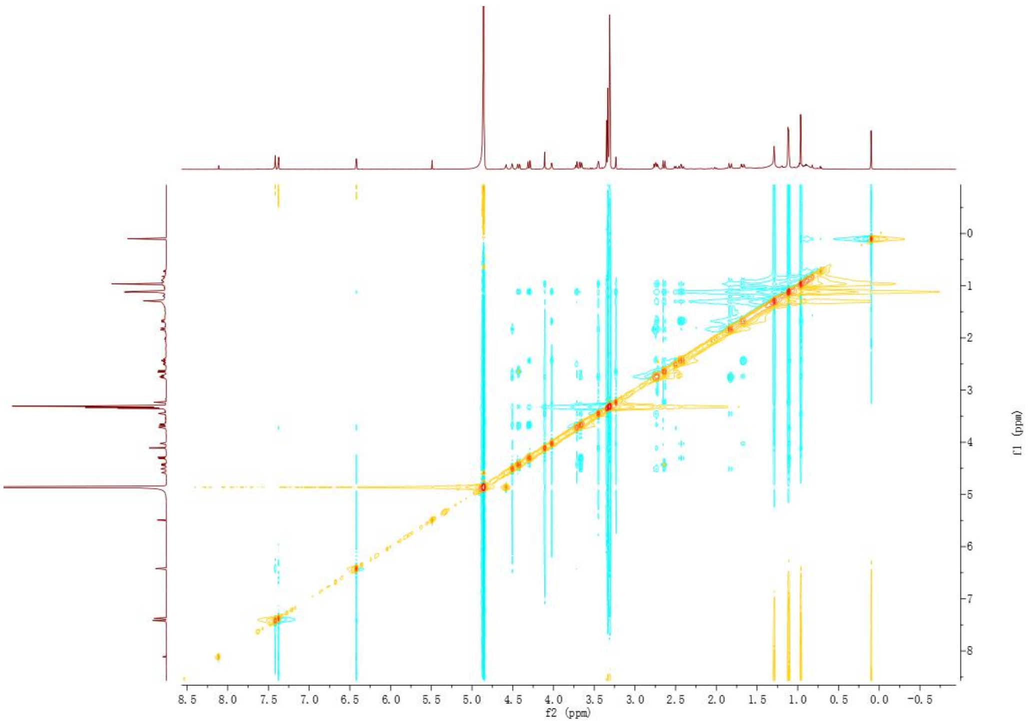

Figure S3 NMR analysis of 29-*Oxo*-12-dehydroneoazedarachin D (**3**) (A, IR; B, HR-ESI-MS; C, ^{1}H NMR; D, ^{13}C NMR; E, DEPT135° ; F, HSQC; G, HMBC; H, ^{1}H-^{1}H COSY; I, NOESY)

Figure S3A

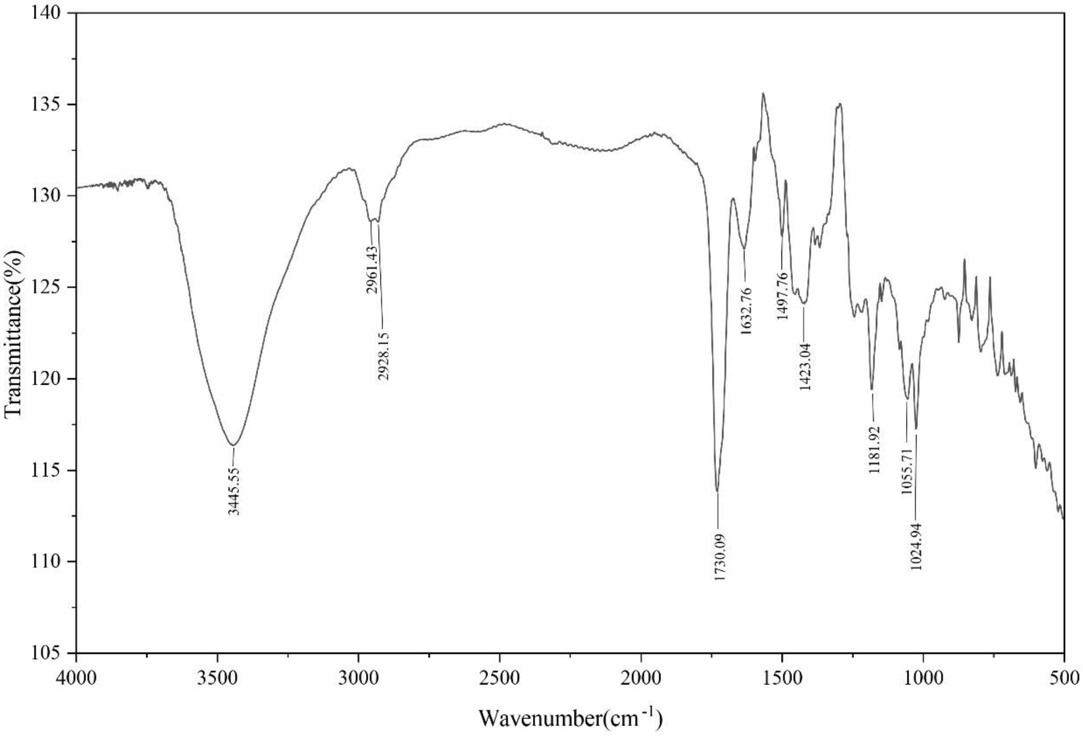

Figure S3B

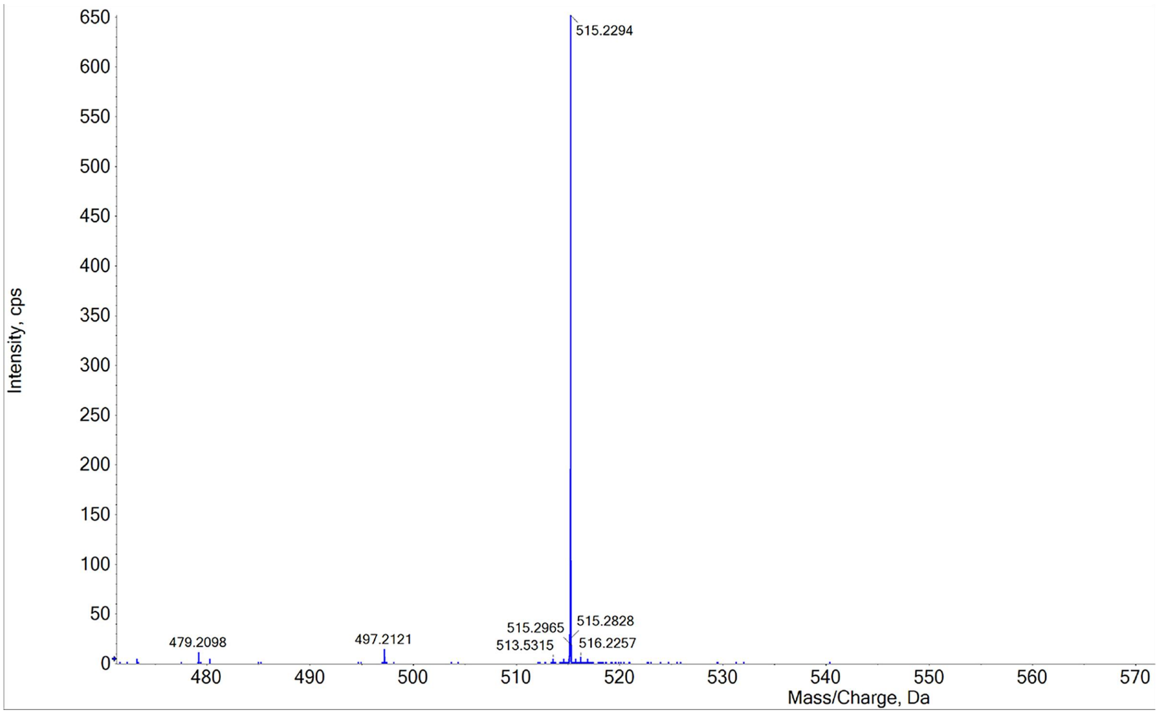

Figure S3C

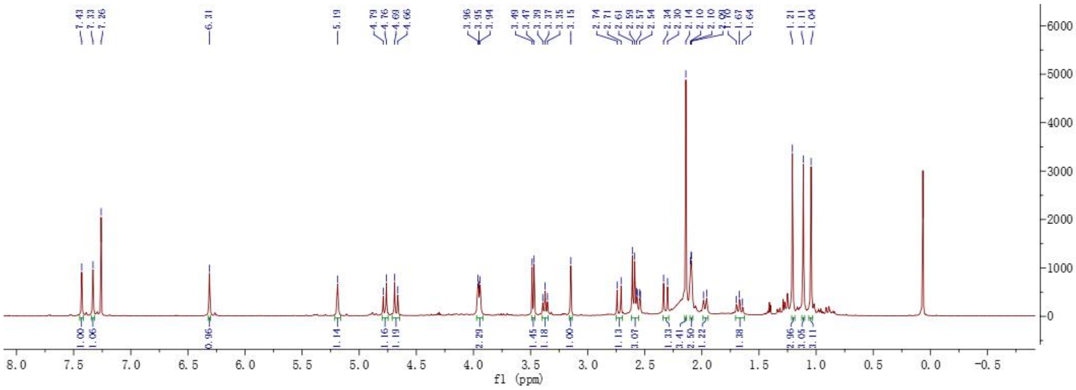

Figure S3D

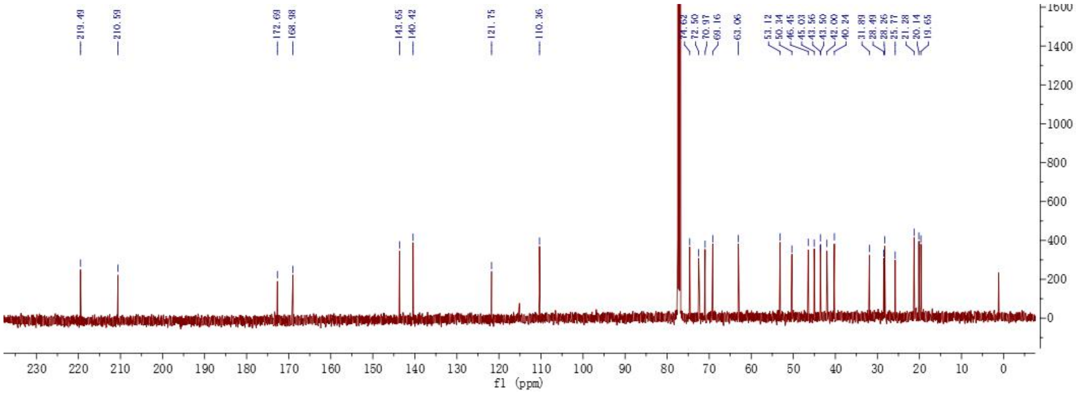

Figure S3E

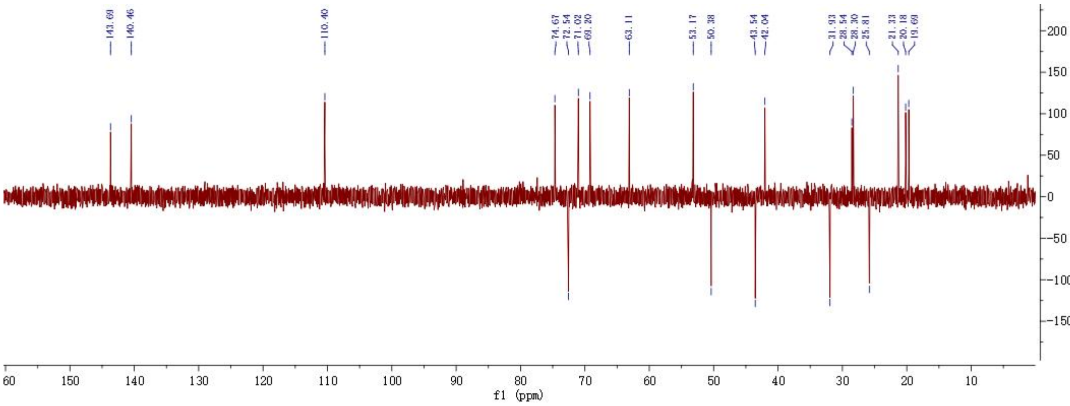

Figure S3F

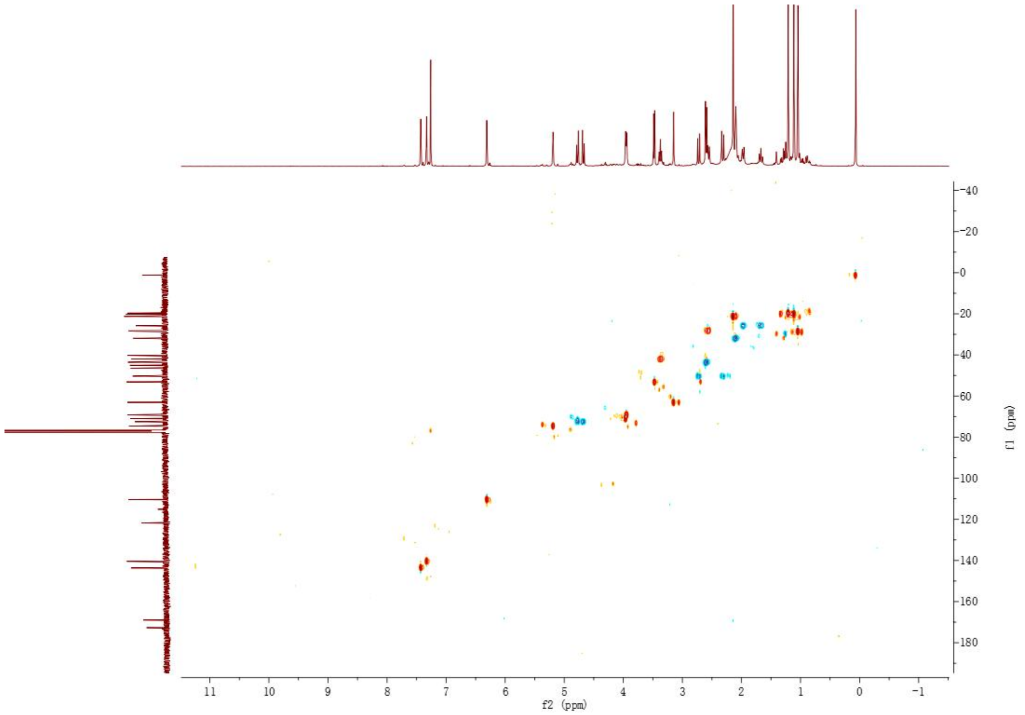

Figure S3G

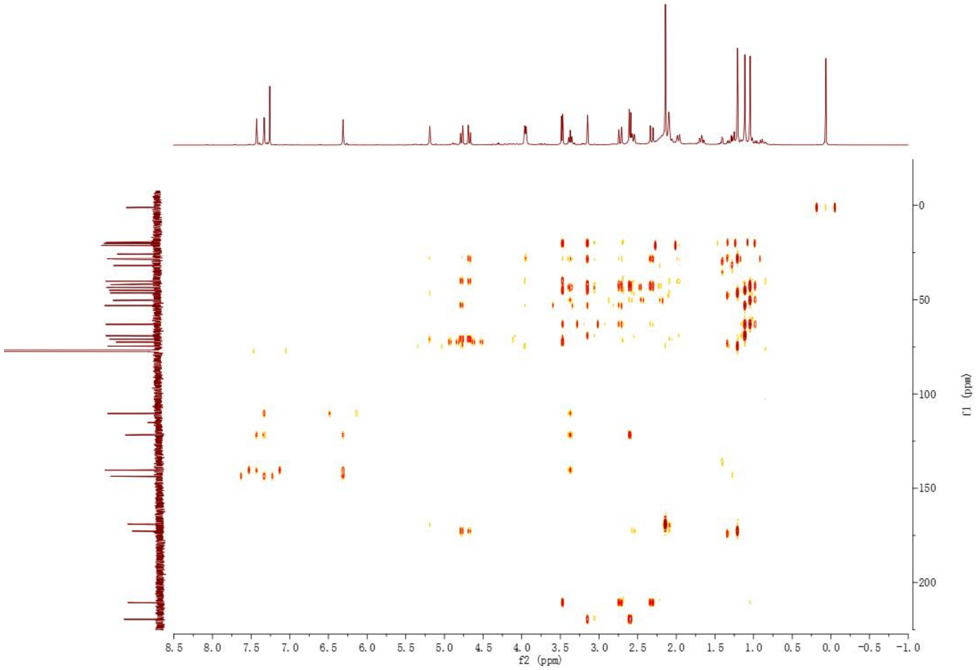

Figure S3H

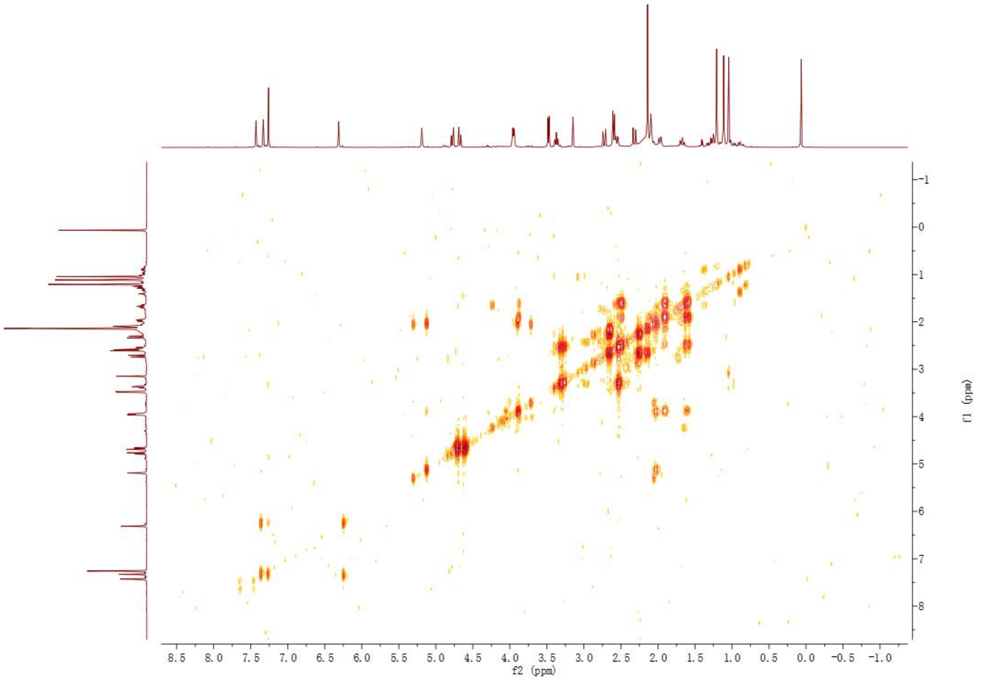

Figure S3I

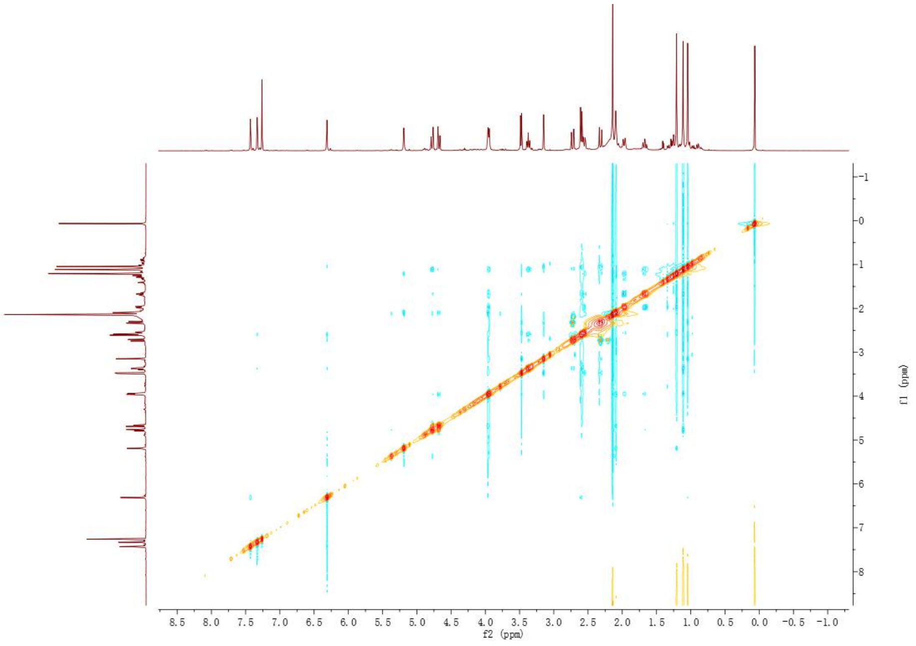

Supplement: Supplementary file 1 [file antioxidants-15-00562-s001.zip › Supplementary Material S3.pdf]
